# Phenotypic screen and transcriptomics approach complement each other in functional genomics of defensive stink gland physiology

**DOI:** 10.1186/s12864-022-08822-z

**Published:** 2022-08-20

**Authors:** Sabrina Lehmann, Bibi Atika, Daniela Grossmann, Christian Schmitt-Engel, Nadi Strohlein, Upalparna Majumdar, Tobias Richter, Matthias Weißkopf, Salim Ansari, Matthias Teuscher, Muhammad Salim Hakeemi, Jianwei Li, Bernhard Weißbecker, Martin Klingler, Gregor Bucher, Ernst A. Wimmer

**Affiliations:** 1 Johann-Friedrich-Blumenbach-Institute for Zoology and Anthropology, Dept. of Developmental Biology, Georg-August-University Goettingen, GZMB, Ernst-Caspari-Haus, Justus-von-Liebig-Weg 11, Goettingen, 37077 Germany; 2 Johann-Friedrich-Blumenbach-Institute for Zoology and Anthropology, Dept. of Evolutionary Developmental Genetics, Georg-August-University Goettingen, GZMB, Ernst-Caspari-Haus, Justus-von-Liebig-Weg 11, Goettingen, 37077 Germany; 3 Department of Biology, Division of Developmental Biology, Friedrich-Alexander University of Erlangen-Nürnberg (FAU), 91058 Erlangen, Germany; 4 Institute for Developmental Biology, University of Cologne, 50674 Cologne, Germany; 5 Present address: The Pirbright Institute, Ash Road, PirbrightWoking, GU24 0NF United Kingdom; 6 Dept. of Forest Zoology and Forest Conservation, Georg-August-University Goettingen, Buesgen-Institute, Buesgenweg 3, 37077 Goettingen, Germany

**Keywords:** Chemical ecology, Genome-wide, iBeetle, Odoriferous glands, RNA interference, RNAseq *Tribolium castaneum*

## Abstract

**Background:**

Functional genomics uses unbiased systematic genome-wide gene disruption or analyzes natural variations such as gene expression profiles of different tissues from multicellular organisms to link gene functions to particular phenotypes. Functional genomics approaches are of particular importance to identify large sets of genes that are specifically important for a particular biological process beyond known candidate genes, or when the process has not been studied with genetic methods before.

**Results:**

Here, we present a large set of genes whose disruption interferes with the function of the odoriferous defensive stink glands of the red flour beetle *Tribolium castaneum*. This gene set is the result of a large-scale systematic phenotypic screen using RNA interference applied in a genome-wide forward genetics manner. In this first-pass screen, 130 genes were identified, of which 69 genes could be confirmed to cause phenotypic changes in the glands upon knock-down, which vary from necrotic tissue and irregular reservoir size to irregular color or separation of the secreted gland compounds. Gene ontology analysis revealed that many of those genes are encoding enzymes (peptidases and cytochromes P450) as well as proteins involved in membrane trafficking with an enrichment in lysosome and mineral absorption pathways. The knock-down of 13 genes caused specifically a strong reduction of para-benzoquinones in the gland reservoirs, suggesting a specific function in the synthesis of these toxic compounds. Only 14 of the 69 confirmed gland genes are differentially overexpressed in stink gland tissue and thus could have been detected in a transcriptome-based analysis. However, only one out of eight genes identified by a transcriptomics approach known to cause phenotypic changes of the glands upon knock-down was recognized by this phenotypic screen, indicating the limitation of such a non-redundant first-pass screen.

**Conclusion:**

Our results indicate the importance of combining diverse and independent methodologies to identify genes necessary for the function of a certain biological tissue, as the different approaches do not deliver redundant results but rather complement each other. The presented phenotypic screen together with a transcriptomics approach are now providing a set of close to hundred genes important for odoriferous defensive stink gland physiology in beetles.

**Supplementary Information:**

The online version contains supplementary material available at 10.1186/s12864-022-08822-z.

## Background

Functional genomics uses unbiased genome-wide approaches to identify the biological function of genes based on high-throughput or large-scale experimental methodologies [1]. To link gene functions to particular phenotypes, functional genomics uses systematic gene disruption or analyzes natural variations such as gene expression profiles of different tissues from multicellular organisms [2]. Therefore, functional genomics approaches make it possible, to identify sets of genes that are specifically important for the development and physiology of a certain tissue. Such approaches are of particular importance to complete the knowledge beyond candidate genes from the classic genetic models, or when a certain tissue has not yet been studied with genetic methodologies before.

Beetles and ants are the most prolific producers of defensive substances [3], which are usually multifunctional and operate as repellents, surfactants, antimicrobics, or toxicants against a large array of potential target organisms [4]. Many Coleoptera biosynthesize and store their defensive compounds in complex glands and release them by controlled opening of the gland reservoirs [5]. In *Tribolium* beetles (Coleoptera: Tenebrionide), odoriferous defensive stink glands [6] are present in pairs in the caudal abdomen (posterior, abdominal, or pygidial glands) and in the prothorax (anterior, thoracic, or prothoracic glands). The glands are composed of two types of secretory units with particular vesicular organelles, tubules, duct, a reservoir and respective muscles [6–8]. In the glands of Tenebrionid beetles, highly reactive, unstable, and toxic para-benzoquinone compounds are produced in large amounts [5, 6, 8–12] to condition the flour they live in to become unusable for competing microorganisms [13]. *Tribolium* beetle secretions were noticed, since conditioned flour turns pink [14] and becomes unusable as well as hazardous to human health [15, 16]. Major secretion components besides the toxic para-benzoquinones are 1-alkenes also called terminal olefins [17–21]. These represent extremely versatile chemical intermediates and thus serve as important products with direct application in the production of biofuels or other industrial chemicals such as plasticizers, emulsifiers, or biodegradable surfactants [22].

For *Tribolium* stink glands, more than fifty years ago, histological and biochemical techniques revealed some basic insights into the process of toxic para-benzoquinone production [8] and about thirty years ago into terminal olefin biosynthesis [20]. However, no more details on how the different enzymatic activities are localized to the different compartments have so far been obtained. Howard already pointed out almost thirty years ago [23], that molecular genetics has so far dealt little with this topic despite its great potential to help to better understand semiochemical and defensive compound secretion. Even today not much is known about the genes that are required for regulating and executing the production of defensive secretions or for self-protection mechanisms against auto-intoxication by the defensive compounds. This is probably because the main genetic insect model, *Drosophila melanogaster*, does not have such glands. Recently, however, the red flour beetle, *T. castaneum*, has emerged as a genetic model organism to study development, physiology and coleopteran pest biology with an array of tools available for functional genetic work [24]. *T. castaneum* carries defensive glands and at least some genetic data on gland physiology come from this species with three mutant strains carrying visibly gland phenotypes: *melanotic stink glands* (*msg*; [25]), *tar* [26], and *box* (*A*^*box*^; [27]). The mutations affect para-benzoquinone secretion and result in modification of the substances contained in the reservoirs. Moreover, a first functional genomics approach based on transcriptome analysis identified 77 genes in this species that were 64 × higher expressed in the gland tissue compared to an abdominal control tissue. Of these, 71 genes were functionally analysed by RNA interference-mediated (RNAi) gene knock-down in respect to their necessity for gland morphology and function [28]. In these study, phenotypic changes in morphology as well as gland volatile content could be observed.

The functional genetic tools in *T. castaneum* include forward genetics based on insertional mutagenesis [29], transgene-based miss-expression systems [30, 31], a fully annotated genome sequence [32–34], as well as systemic RNAi [35, 36]. The efficient use of RNAi in this beetle has allowed the development of this approach into a forward genetics application to perform an unbiased genome-wide large-scale phenotypic screen (iBeetle screen) to identify gene functions in embryonic and postembryonic development as well as cell biology and physiology [37, 38]. In this screen, knock-down beetles and their progeny were systematically analyzed for phenotypic changes including their stink glands and methodically documented in iBeetle-Base [39, 40]. With the iBeetle screen, we were for the first time able to identify a large set of genes required for stink gland function directly based on phenotypes. We used the iBeetle-Base documentation of this first-pass screen to re-screen all 130 genes identified for potentially affecting gland formation and function. For 69 of these genes, we could validate that the knock-down indeed had an effect on the gland phenotype. Here, we present the genes of this unbiased phenotypic screen with their phenotypes and compare the results with the previously identified stink gland gene set using transcriptome analysis based on tissue-specific RNAseq analysis [28].

## Results and discussion

### iBeetle: Large-scale genome-wide phenotypic screen

iBeetle stands for a large-scale systemic RNAi-based screen using the red flour beetle, *T. castaneum*, as a screening platform. Double-stranded RNA injections were performed in larval or pupal stages and the phenotypic effects scored at multiple levels [37, 38]. In the 1st and 2nd phase of iBeetle, a total of around 8500 gene models were screened for their function by systematic gene knock-down, which corresponds to slightly more than 50% of the currently annotated genes in this organism [34]. The obtained phenotypes were documented in iBeetle-Base [39, 40] by nine different screeners, who checked systematically for phenotypes in respect to embryonic and postembryonic development as well as cell biology and physiology. The phenotypic analysis included the detection of visible morphological changes affecting the defensive stink glands of this beetle. However, without a detailed investigation by systematic dissection and a clear expectation what gland phenotypes could be detected through the adult cuticle, the phenotype descriptions annotated in iBeetle-Base were quite variable and not standardized (Additional file 1: Supplementary Table S1). Thus, iBeetle serves as a first-pass screen to identify potentially interesting genes for a particular biological process. These genes need then to be verified by a detailed re-screening process with a particular focus on the tissue of interest. For 130 genes, corresponding to about 1,5% of the analyzed gene models, phenotypic changes in gland morphology were annotated in iBeetle-Base. These were subject to a re-screen procedure based again on RNAi, and for 69 of them (53%), a knock-down gland phenotype could be detected (Additional file 1: Supplementary Table S1). In 60 cases, the originally iBeetle-identified phenotype could be confirmed, whereas in nine cases (all from the 2nd iBeetle phase) a gland phenotype different from the originally described one was detected (Table 1). While the iBeetle screen was using a transgenic enhancer trap strain (Pig-19) [41], the re-screen was performed in the wild type San Bernadino strain. The difference in the observed phenotypes might thus be caused by strain-specific differences, which have previously been observed in *T. castaneum* RNAi-induced phenotypes [42].

**Table 1:** Comparison of iBeetle phenotypic screen with transcriptome analysis

		FC ≥ 2
iBeetle annotated gland phenotypes	130	17
- re-screen confirmed iBeetle gland phenotypes	60	12
- re-screen observed different gland phenotypes	9	2
**Re-screen identified gland phenotypes**	**69**	**14**

Indicated are the number of genes

FC ≥ 2: expression in glands twofold higher than in control tissues [28]

### Morphological knock-down phenotypes of *Tribolium castaneum* stink glands

**Fig. 1:**
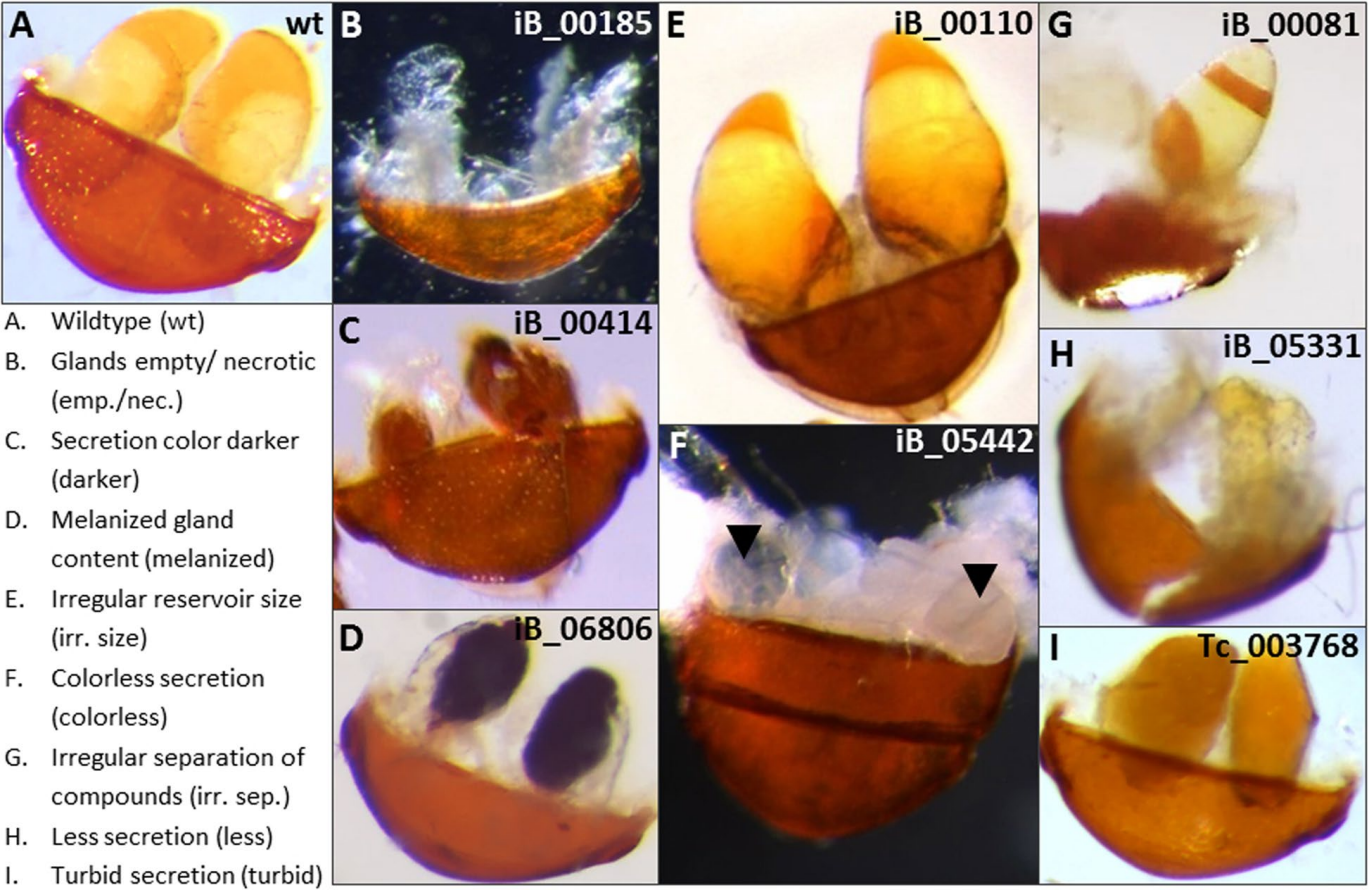
Visible morphological stink gland phenotypes identified in specific gene knock-downs. Morphologies differing from wild type (**A**) were categorized into seven groups: glands empty and/or necrotic (**B**); color of secretion darker (**C**); color of secretion melanized (**D**); reservoir size irregular (**E**); colorless secretion (**F**); irregular separation of gland compounds (**G**); containing less secretion (**H**); or turbid secretion (**I**), a phenotype that was not observed in the iBeetle screen or re-screen, but was detected in the knock-down of one of the transcriptomics-identified genes (Tc_003768) and originally described as “condensed” [28]. The iBeetle numbers of the representative gene knock downs the pictures were taken of are indicated

For the re-screen of all 130 genes with an annotated gland phenotype, the visible morphological phenotypes were then categorized into seven groups (Fig. 1): glands empty and/or necrotic (Fig. 1B); reservoir size irregular (increased or decreased, Fig. 1E) or containing less secretion (Fig. 1H); color of the secretion either darker (Fig. 1C), melanized (Fig. 1D), colorless (Fig. 1F), or showed an irregular separation of the gland compounds (Fig. 1G). An additional gland phenotype, that was not observed in the iBeetle screen or re-screen, is turbid secretion (Fig. 1I), which was detected in the knock-down of one of the transcriptomics-identified genes (Tc_003768; GT12) and originally described as “condensed” [28]. The knock-down of this particular gene causes a rare alkene-less phenotype not affecting the benzoquinone production [28], which has so far not been observed for any other gene knock-down. The comparison of the originally described gland phenotypes in the iBeetle screen with the categorized phenotypes of the re-screen is provided in Additional file 1: Supplementary Table 1. For the 69 genes with a re-screen confirmed gland phenotype, the gland morphology category is also indicated in Additional files 2 and 3: Supplementary Tables S2 and S3.

For 61 genes, the originally annotated phenotype in iBeetle-Base could not be reproduced even with injections of higher concentrations of dsRNA. The majority of the annotated stink gland phenotypes for these genes are hard to identify (‘less secretion’, ‘irregular reservoir size’, and ‘secretion color darker’), as also in wild type beetles, stink gland secretions display natural variation in the degree of filling, color, and shape. Therefore, it is highly likely that these genes were assigned as false-positives in the first-pass iBeetle screen that needs re-screening for confirmation.

### Gene ontology analysis of genes required for stink gland physiology

**Fig. 2:**
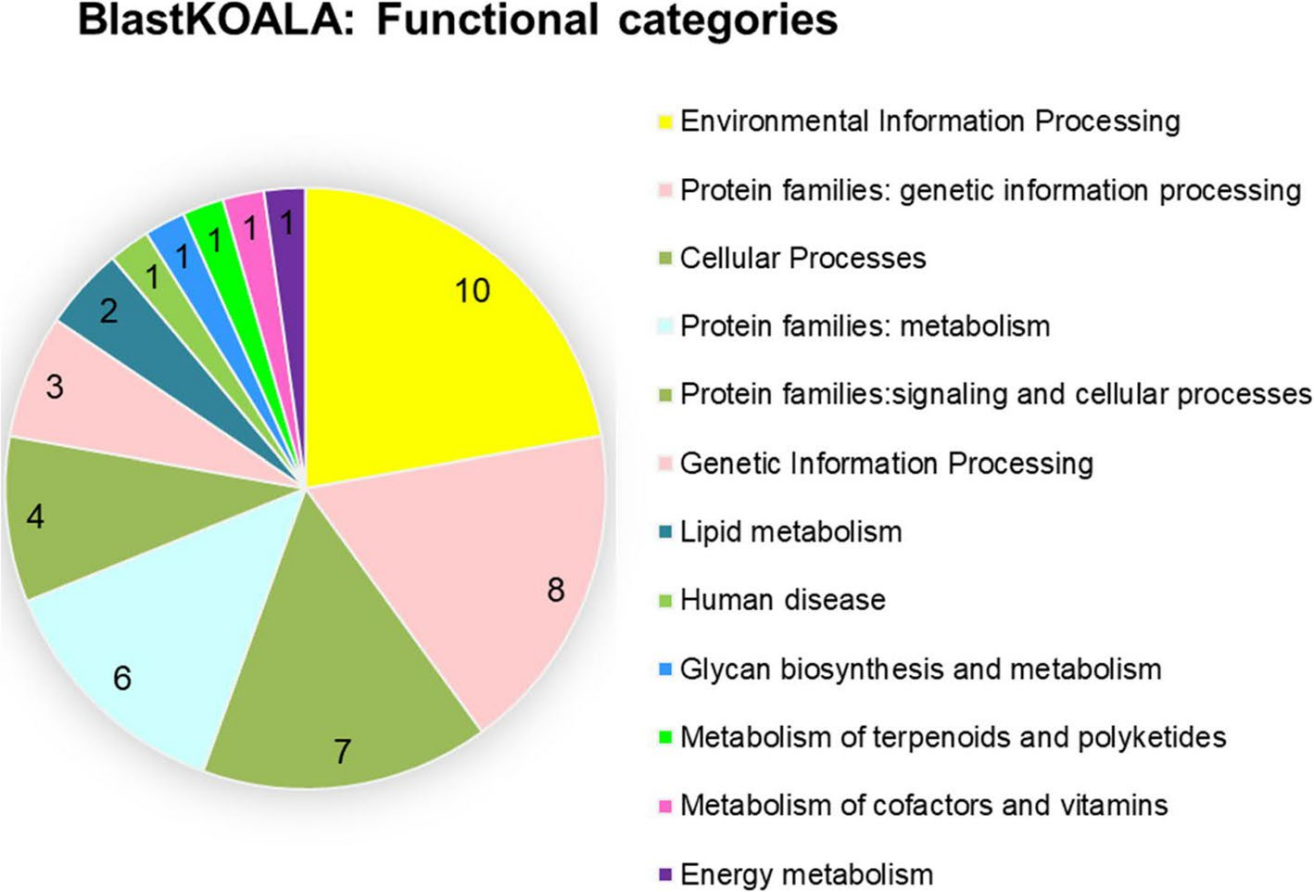
Gene ontology analyses of confirmed gland genes. BlastKOALA KEGG pathway analysis was performed on taxonomy group Eukaryotes, Animals, Arthropods (Taxonomy ID 7070) searching KEGG databases family_eukaryotes.pep + genus_prokaryotes.pep. 45 proteins were analyzed and put into twelve functional categories

To identify the molecular function of the 69 functionally rescreen-confirmed gland genes, the nucleotide or amino acid conserved domains were identified using the respective National Center for Biotechnology Information (NCBI) online search tool [43] and homologs were screened for specifically in *D. melanogaster* and the entire NCBI nucleotide collection database. The results are provided in Additional file 2: Supplementary Table S2. To further analyze gene ontologies of those 69 genes and identify potential metabolic pathways, we performed ShinyGO [44], BlastKOALA [45] and eggNOG-mapper [46] analyses. ShinyGO could analyze 68 genes (Additional file 2: Supplementary Table S2) and identified “lysosome (ko04142)” as the only significantly enriched KEGG pathway (Additional file 4: Supplementary Figure S1). In the BlastKOALA KEGG pathway analysis, 45 genes could be analyzed and were put into twelve functional categories with most genes assigned to environmental information processing, genetic information processing, cellular processes, and metabolism (Fig. 2). Also in this analysis, the cellular process lysosome (ko04142) was identified with the same four genes as well as the organismal system pathway “mineral absorption (ko04978)” covered by three genes (Additional file 5: Supplementary Figure S2). One of these genes, iB-02517, encodes copper-transporting ATPase-I (ATP7), which had already been reported for *Tribolium* gland function [37]. While ATP7 does not belong to the ATP-binding cassette (ABC) transporters, it is still interesting to note that ATP-binding transporters have previously been shown to be involved in defensive glands of leaf beetles for sequestration of phytochemicals [47, 48]. eggNOG-mapper analyzed 65 out of the 69 provided query proteins (Additional file 2: Supplementary Table S2). Similar to the BlastKOALA Brite analysis (Additional file 6: Supplementary Figure S3A), also the eggNOG-mapper Brite analysis (Additional file 6: Supplementary Figure S3B) identified many enzymes including kinases, phosphatases, glycosyltransferases, peptidases, and cytochrome P450s as well as proteins involved in membrane trafficking (Additional file 6: Supplementary Figure S3). Altogether, many genes identified are involved in Golgi apparatus, exosome and lysosome function as expected for a secretory tissue.

### Changes in stink gland volatile compounds of gland gene knock-downs

**Fig. 3:**
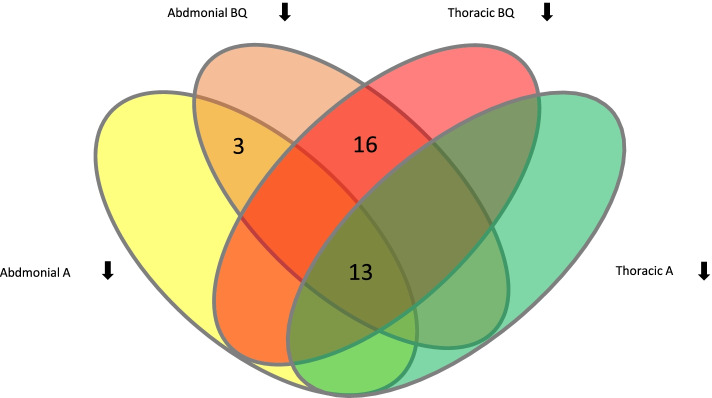
Changes of stink gland volatile compounds in knock-down beetles of iBeetle-confirmed genes. For 13 genes, strongly reduced levels of benzoquinones (BQ) and alkenes (A) in both abdominal and thoracic glands were detected, while for 16 genes strong reduction of BQ in abdominal and thoracic glands, and for 3 genes strong reduction of BQ only in abdominal gland were observed. BQ represents benzoquinones 2-Methyl-1,4-benzoquinone (MBQ) and 2-Ethyl-1,4-benzoquinone (EBQ), while A represents the two alkenes 1-Pentadecene (1-C15) and 1-Heptadecene (1-C17)

To examine whether the knockdown of the 69 re-screen-identified genes not only caused a visible morphological phenotype but also a change in gland contents, we applied gas chromatography–mass spectrometry to analyze the volatile compounds of the gland secretions independently for the thoracic and abdominal glands. In wild type beetles, the volatile secretion composition is similar in males and females [28], with the four main volatiles corresponding to two para-benzoquinones, 2-Methyl-1,4-benzoquinone (MBQ) and 2-Ethyl-1,4-benzoquinone (EBQ), and two alkenes, 1-Pentadecene (1-C15) and 1-Heptadecene (1-C17). In the different gene knock-downs, the abundances of the four main volatiles were altered to different degrees, reaching from higher than wild type levels, to no alteration, and down to undetectable levels of all or specific compounds (Additional file 3: Supplementary Table S3). The two para-benzoquinones or the two alkenes were usually affected together, whereas the production of the two compound groups seems to be independent. Of the 69 genes analyzed, we found in total that 32 showed strong changes (≥ 50% reduction) in at least one type of volatile compounds (para-benzoquinones or alkenes) and one type of gland (Fig. 3). Very strong reductions of both benzoquinones and alkenes in thoracic and abdominal glands were observed in the knock-down of 13 genes, which, however, gave rise to very different morphological gland phenotypes (colorless, irregular size, empty/necrotic, darker, melanized). This indicates that the lack of volatile compounds can be the result of very different causes.

Interestingly, 16 gene knock-downs caused specifically a strong reduction of para-benzoquinones levels only, suggesting a specific function in the synthesis of these compounds. In contrast, none of the 69 gene knock-downs led to a specific reduction in alkene production only. In three cases, reductions of volatile levels were observed to be gland-type specific restricted to the abdominal gland only. However, it should be noted that measurements have been done only once with a small number of individuals and all effects will have to be confirmed in future experiments. For three genes (iB_04797, iB_05441, iB_09043), whose knock-down caused the morphological phenotype ‘colorless’, also a lack of benzoquinones for both sexes and in both types of glands was observed, linking the yellowish color in wild type stink glands to the presence of para-benzoquinones in the secretions. Besides that, no direct correlation between the visible morphological phenotype and the secretion volatile compound phenotype could be recognized (Additional file 3: Supplementary Table S3).

### Transcriptome-based expression levels of iBeetle-identified gland genes

To examine, whether the phenotypically detected genes during the iBeetle screen could have also been identified by a functional genomics approach based on transcriptomics analysis, we identified their expression levels in the published *Tribolium* stink gland transcriptome [28]. Out of the 130 originally identified genes, only 17 have a more than two times higher expression in the gland tissue (Table 1 and Additional file 1: Supplementary Table 1) and about a third have even a clearly reduced level of expression in the gland tissue compared to a control tissue (Additional file 3: Supplementary Table 3). Thus, the phenotypic screen can also identify genes that are not specifically active in the glands but also in other tissues, but are still necessary for the function of the glands. Of the 69 re-screen-confirmed gland genes, only 14 genes (20%; Table 1), and of the 32 gland genes with a strong secretion phenotype (Fig. 2), only seven genes show a two-fold or higher (FC ≥ 2) expression in the gland tissue (Additional file 7: Supplementary Table S4). This indicates that about 80% of the genes with a function in stink gland physiology will be missed using only differential gene expression data to select candidate genes.

### Transcriptome-identified gland genes covered by the iBeetle screen

**Fig. 4:**
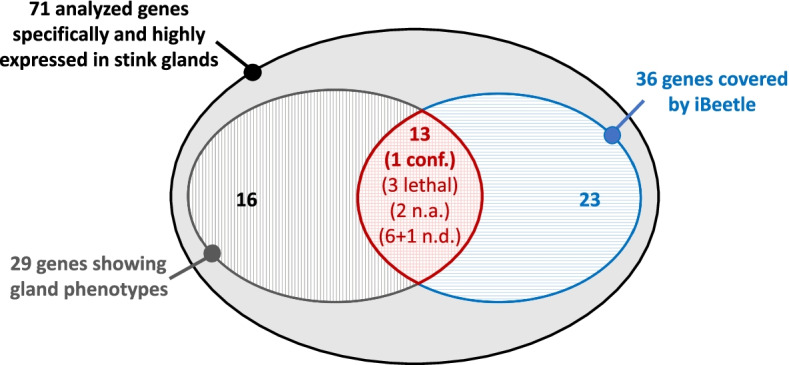
Transcriptomics-identified genes analyzed in iBeetle phenotypic screen. Of 71 genes identified by a transcriptomics approach to be highly and specifically expressed in gland tissue, 29 genes showed a gland phenotype in knock-down situations [28]. Of the 71 genes, 36 genes were analyzed during the 1st and 2nd phase of the iBeetle screen. 13 thereof had shown gland phenotypes in the transcriptomics-based analysis [28], but only one was confirmed (conf.) in the iBeetle screen. Two genes were not analyzed (n.a.) for stink gland phenotypes as they were only part of the pupal screen of the 1st phase) and seven (6 + 1) genes non detected (n.d.), of which at least one should have been detectable due an easily visible strong melanized gland content phenotype

In the odoriferous defensive stink gland transcriptome data, Li et al. [28] identified 77 genes that are highly and specifically expressed in the stink glands (FC ≥ 64). 71 of them were analyzed in gene knock-downs for visible morphological and secretion volatile stink gland phenotypes. Only 29 of the 71 genes showed altered morphological and secretion volatile phenotypes in the stink glands (Additional file 8: Supplementary Table S5) [28]. Out of the 71 genes identified by transcriptomics, 36 were also analyzed during the first or 2nd phase of the iBeetle screen (Fig. 4), with 13 genes belonging to the fraction showing varied phenotypes in the transcriptomics-based knock-down analysis. However, from these 13, only one gene (iB_09413), which caused a melanized phenotype, was confirmedly identified (conf.) also in the iBeetle screen. Two (iB_5763 and iB_5847) were not analyzed (n.a.), since they belonged to the 800 genes that were not covered in the larval screen of the 1st phase of iBeetle and also not covered in the pupal screen of the 2^nd^ phase, since they were already covered in the 1^st^ phase pupal screen, which however did not cover the stink gland analysis. For three genes covered in the 1st phase of iBeetle, the larval injection screen caused a lethal phenotype making a comparison to the functional analysis of the transcriptome-identified genes impossible, since these were analyzed by pupal injections [28]. For seven genes, the gland phenotypes were not detected (n.d.) in the iBeetle screen despite that fact that they were analyzed. For six of these genes, the phenotypes are hard to detect (less secretion, colorless, turbid, empty/necrotic; Additional file 8: Supplementary Table S5) and could have easily been missed by the diverse screeners of iBeetle, who scored the gland phenotype without dissection of the glands just through the cuticle when observing the injected adult beetles. However, one gene (iB_07205) causes a melanized phenotype that should have been detected in the iBeetle screen. This might have simple be missed by the screener in a large scale, first-pass screen without repetition. Or it might have been due to the injection of different strains, as RNAi knock-down phenotypes have previously been observed to be potentially strain-specific in *T. castaneum* [42]. In summary, the iBeetle screen identified only one out of eight gland genes that had been identified in the transcriptomics-based approach by Li et al. [28] and were functionally analyzed during the iBeetle screen (Fig. 4). This indicates that a large, first-pass, multipurpose screen carried out by multiple screeners with diverse interests such as iBeetle [37, 38] can only serve as a starting point for the identification of genes acting in a certain biological process not studied by genetic means before.

## Conclusion

*T. castaneum* is a significant worldwide pest beetle of stored grains. It produces and releases defensive secretions acting as toxic, repellent, bacteriostatic, and fungistatic oils containing substituted para-benzoquinone compounds. To identify a large set of genes that are specifically important for such a particular tissue that had not been studied with genetic methods before, our present study indicates that one needs to combine several diverse functional genomic approaches. In a previous transcriptomics-based analysis [28], 77 genes were identified to be highly and specifically expressed in the gland tissue and for 29 of them it could be shown, that a knock-down causes a visible morphological gland phenotype. In our present study, we could identify 69 genes by a phenotypic screen to cause such altered glands. Only one gene was identified in both screens, indicating the different approaches do not deliver redundant results but rather complement each other. It was expected that a phenotypic screen would also identify genes that are not specifically expressed in the stink glands, since other tissues might provide necessary precursor substances to be further processed in the glands [8]. However, the fact that only one out of eight genes functionally analyzed in both approaches was identified in the phenotypic screen indicates that this screen probably recognized only a small subset of genes involved in stink gland function. Both functional genomics approaches together are now providing a set of almost hundred genes (97) that have been shown to be necessary for odoriferous defensive stink gland physiology in beetles.

## Methods

### *Tribolium *rearing

*T. castaneum* (Herbst, 1797; Insecta, Coleoptera, Tenebrionidae) strains were reared on organic wheat flour supplemented with 5% yeast powder at 28 °C and 40% relative humidity under constant light. The Beetles were collected from different breeding boxes varying in age (up to three month) and culture density.

### iBeetle 1st phase: screening and rescreening:

In the 1st phase of the iBeetle screen, which covered 5300 genes, potential gland phenotypes were analyzed in the ‘larval screen’ part, in which penultimate larvae (L6) of a specific cross [37] were injected with 1 µg/µl double-stranded RNA of the respective iBeetle fragment (https://ibeetle-base.uni-goettingen.de/) [38, 39]. The glands were then inspected at the adult stage 38 to 41 days after injection for aberrant gland phenotypes. However, the larval screen actually only covered about 4500 genes, resulting in 800 genes that were not screened for involvement in gland function. In iBeetle-Base [39], 57 genes were noted to probably have a function in gland physiology, for which gene fragments were identified that did not overlap with the original iBeetle screen fragment (Additional file 5: Supplementary Table 5). To confirm the knock-down-induced gland phenotype of these genes, double-stranded RNA of non-overlapping fragments were injected at a concentration of 2 to 3 µg/µl into pupae or larvae of the wild type San Bernadino strain. For 32 genes, the iBeetle-identified phenotype could be confirmed (Additional file 1: Supplementary Table 1).

### iBeetle 2nd phase: screening and rescreening

In the 2nd phase of the iBeetle screen, which covered 3200 genes, double-stranded RNA injection of iBeetle fragments (https://ibeetle-base.uni-goettingen.de/) [38, 39] was performed at a concentration of 1 µg/µl into pupae of the PIG-19 strain [41]. Stink gland analysis was carried out at the adult stage 21 days after injection [38]. For the 2nd phase, an additional 73 genes were noted in iBeetle.Base [40] to potentially have a knock-down-mediated altered phenotype. These 73 genes were re-screened by injecting the original iBeetle screen fragment again into pupae of the wild type San Bernadino strain at a concentration of 1 µg/µl. For 28 genes the iBeetle-identified gland phenotype could be confirmed, while for nine genes a gland phenotype could be detected that did not match the originally described phenotype (Additional file 1: Supplementary Table 1). Based on the morphological (Fig. 1; Additional file 1: Supplementary Table S1) and the volatile gland compound phenotype (Fig. 3; Additional file 3: Supplementary Table S3), for 19 genes – 18 confirmed plus one non-matching gland phenotype (iB_07902) – also non-overlapping fragments were identified (Additional file 10: Supplementary Table S7). After double-stranded RNA injection of these at a concentration of 2 µg/µl into wild type San Bernadino strain pupae, the re-screen phenotype could be confirmed for all 19 genes.

### Sequences, genome assemblies, and gene identifiers used

The first two phases of the iBeetle screen started on the knowledge of the draft genome assembly Tcas3.0 [32] and the official gene set (OGS) 2 [33]. The sequences used for the non-overlapping fragments for the re-screens are thus based on Tcas3 (Additional files 9 and 10: Supplementary Tables S6 and S7). The gene function analysis (Additional file 1: Supplementary Table S1) and the gene ontology analysis (Additional file 2: Supplementary Table S2) are based on the current OGS 3 and genome assembly Tcas5.2 [34] with changes in Tc gene numbers between Tcas3 and Tcas5.2 indicated in Additional file 1: Supplementary Table S1. In all tables but one, the genes are sorted by the unique iBeetle number (https://ibeetle-base.uni-goettingen.de/) [38, 39] and cross-referencing to the Tc gene number is provided. Only in Additional file 8: Supplementary Table S5, the genes are listed based on the Tc numbers derived from Tcas3.0, which was the genome assembly used at the time of the transcriptome analysis [28], but annotation notes regarding changes into genome assembly Tcas5.2 are provided. The transcriptome analysis [28] is also the reason, why in Additional file 7: Supplementary Table S4, the iBeetle numbers are referring to the Tc numbers of assembly Tcas3.0.

### Double-stranded RNA synthesis and injection

In the re-screening of the 1st phase of iBeetle, double stranded RNAs were self-designed using the E-RNAi web service of the German cancer research center [49] to identify best fragments without off-targets. The fragments were cloned and the in vitro transcription was performed with the MEGAscript® T7 Kit from Ambion® (Life Technologies GmbH, Darmstadt, Germany, Cat#: AM1334) using the purified PCR product of the respective gene fragment with added T7-RNA Polymerase promoter sites as template DNA. In the re-screening of the 2nd phase of iBeetle, original iB-fragments and non-overlapping fragments at a concentration of 3 μg/μl were ordered from Eupheria Biotech GmbH (Dresden, Germany). The synthesized double stranded RNAs were re-suspended and diluted in injection buffer (10 × stock: 14 mM NaCl, 0.7 mM Na2HPO4 ∙ 2H2O, 0.3 mM KH2PO4, 40 mM KCl) and stored at -20 °C.

Double stranded RNA injections were usually carried out at mid-pupal stage of male and female beetles. Before injection, pupae were incubated for 5 min on ice and then lined up on an adhesive tape placed on a microscope glass slide. Borosilicate glass capillaries (length: 100 mm, outside-diameter: 1 mm, wall thickness: 0.21 mm, Heinemann Labortechnik GmbH, Duderstadt, Germany) were pulled with the Micropipette Puller Model P-2000 (Sutter Instruments, Novato, USA) to generate injection needles. For semiautomatic injections, a FemtoJet®express microinjector (Eppendorf, Hamburg, Germany) was used in combination with a micromanipulator (M1, Helmut Saur Laborbedarf, Reutlingen, Germany). The injection was carried out under a stereomicroscope.

### Phenotypic analysis of knock-down beetle stink glands

In the re-screen, the prothoracic and abdominal stink glands were dissected from wild type and knock-down beetles about three weeks after injection. The gland and reservoir morphology was analyzed under a dissection stereomicroscope (Leica MZ16FA) and exemplary pictures of the diverse phenotypes were taken with a Q-imaging camera (Leica Microsystems GmbH, Wetzlar, Germany). Pictures were processed with Adobe Photoshop CS2 (Fig. 1).

The gland content volatiles from the prothoracic and abdominal gland tissues of knockdown and control (wild type) beetles were independently analyzed by semi-quantitative gas chromatography-mass spectrometry (GC–MS). In the beginning of re-screening in the 1st phase of the iBeetle screen, a mixture of the gland secretions of one female and one male beetle was analyzed, while later on and in the re-screening in the 2nd phase of iBeetle, male and female beetles were analyzed separately. In the 2nd phase, gland secretions of three beetles where prepared and analyzed together for each gene and sex. The abdominal and prothoracic glands were collected from ten day old adult beetles and homogenized in 50 μl (one beetle) or 100 μl (three beetles) methanol (Merck Millipore KGaA, Darmstadt, Germany). The processed gland tissue samples were stored on -20 °C before the GC–MS analysis, which was performed within 48 h. Per sample,1 μl was loaded by a split injector into a GC–MS system composed of a gas chromatograph (6890 N Network GC System, Agilent Technologies, Santa Clara, USA) and a mass spectrometer (5973 Network Mass Selective Detector, Agilent Technologies, Santa Clara, USA) connected to a MultiPurposeSampler (MPS, Gerstel, Mülheim, Germany). The GC–MS data were analyzed by the MSD ChemStation D.02.00.275 software (Agilent Technologies, Santa Clara, USA). The volatile secretion compounds were identified with the NIST 2008 and Wiley 9th edition databases (National Institute for Standards and Technology, Gaitherburg, USA; Wiley, Hoboken, USA). The calculations of semi-quantitative analysis of volatile gland secretion substances and comparative chromatograms display were done in Microsoft Excel in both wild type and knock-down situations. For these analyses, first the mean values of abundances of wild type beetle gland chemicals (buffer injected) were determined and set as 100%. Subsequently the relative alterations in secretion substances in gene knock down beetle glands in relation to the wild type mean were calculated in percent, in which values > 100% indicate an increase of the respective substance in the knockdown glands compared to the wild type and values < 100% identify a corresponding reduction. Of the gland volatiles, the two most abundant para-benzoquinones (2-methyl-1,4-benzoquinone and 2-ethyl-1,4-benzoquinone) and the two most abundant alkenes (1-pentadecene and 1-heptadecene) were analyzed (Additional file 3: Supplementary Table S3 and Fig. 3).

### Homolog search, gene ontology, and identification of conserved domains

The encoded proteins of the 69 candidate genes were screened with BLASTp against the *Tribolium* genome assembly Tcas5.2 [50] and with tBLASTn against the *D. melanogaster* genome and the entire NCBI nucleotide collection database. Moreover, nucleotide or amino acid conserved domains were identified using the respective NCBI online search tool [43]. To analyze gene ontologies of those 69 genes and identify potential metabolic pathways, we performed ShinyGO [44], BlastKOALA [45], and eggNOG-mapper [46] analyses. For ShinyGo the respective 69 Tc numbers (TC000379; TC000476; TC000504; TC000885; TC002616; TC006408; TC033883; TC011075; TC012387; TC014494; TC014520; TC015095; TC015165; TC015203; TC015379; TC015811; TC030914; TC030950; TC011288; TC032251; TC002723; TC008303; TC031247; TC002074; TC032367; TC004129; TC005167; TC008047; TC031200; TC009877; TC010251; TC034419; TC012539; TC034399; TC012828; TC012834; TC033122; TC032992; TC033471; TC014887; TC015905; TC000393; TC003827; TC009459; TC001243; TC001275; TC001376; TC032964; TC033206; TC015328; TC015537; TC033755; TC006177; TC016314; TC033022; TC014482; TC008912; TC006363; TC005389; TC005306; TC003116; TC014025; TC014033; TC013627; TC014774; TC033320; TC015429; TC015547; TC014985) were entered online (http://bioinformatics.sdstate.edu/go/) to search the KEGG pathway data base for the species *Tribolium castaneum* (FDR cut off 0.05). For BlastKOALA and eggNOG-mapper the 69 encoded protein sequences were provided in FASTA format (Additional File 11 FASTA file 69 proteins) and entered online https://www.kegg.jp/blastkoala/ or http://eggnog-mapper.embl.de/, respectively. The respective results are provided in Additional file 2: Supplementary Table S2. To generate the column chart for Additional file 6: Supplementary Figure S3B, the KEGG orthologies derived from the eggNOG analysis were summarized online (https://www.genome.jp/kegg/ko.html) using the KEGG ORTHOLOGY (KO) Database [51].

## Supplementary Information

**Additional file 1: Supplementary Table S1.** iBeetle-identified genes involved in defensive stink gland function. The phenotype recognized in the first-pass iBeetle screen and annotated in iBeetle-Base (original detection in 1st or 2nd phase indicated) is compared to the categorized (Fig. 1) phenoytpes of the re-secreen, with phenotypes unchanged form wild type declared as ‘non detected’ (n.d.). The 60 re-screen-confirmed gland phenotypes are indicated in bold. Changes in annotations between Tcas3.0 and Tcas5.2 are provided in brackets. Gland-specific higher expression (FC ≥ 2; Additional file 3: Table S3) is indicted by an “x”.

**Additional file 2: Supplementary Table S2.** Gene ontology of iBeetle-identified genes affecting stink function (separate, sortable Excel file). BLASTp against the *Tribolium *genome assembly Tcas5.2 [50] was used to identified the respective beetle genes and tBLASTn was used against the *D. melanogaster *genome and the entire NCBI nucleotide collection database. In addition, gene ontology analyses were carried out using ShinyGO [44], BlastKOALA [45], and eggNOG-mapper [46]. In ShinyGO, n.a. specifies that one gene (iB-02716) was not analyzed, whereas * indicates the four genes (iB-02516, iB-05119, iB-09043, iB-09239) involved in lysosome function. In BlastKOALA, 24 genes were not analyzed, while in eggNOG-mapper only for four genes (iB-03552, iB-04420, iB-07361, iB-08861) no orthologs were identified. In eggNOG, for one gene (iB_04702) the closest seed ortholog was from the African clawed frog (*Xenopus laevis*)**, while for one gene (iB_08184) it was from the purple sea urchin (*Stronglylocentrotus purpuratus*)***. For all other genes, *T.castaneum *genes were used as seed orthologs by eggNOGG, with 13 genes identified by former Tc-numbers (indicated in column C in addition).

**Additional file 3: Supplementary Table S3.** GC-MS analysis of stink gland volatile compounds of iBeetle-confirmed genes in knock-down beetles (separate, sortable Excel file). Analyzed substances were 2-Methyl-1,4-benzoquinone (MBQ) and 2-Ethyl-1,4-benzoquinone (EBQ), as well as 1-Pentadecene (1-C15) and 1-Heptadecene (1-C17). **Bold** iB numbers indicate that the originally annotated phenotype in iBeetle-Base was confirmed in the re-screen. *Italics *indicate that the gene expression in glands was twofold higher (FC ≥ 2) than in control tissues as identified in a previous study [28]. Reference to the morphological gland phenotype is given in column C. Column T indicates the color code for extraction of the data to generate Figure 3.

**Additional file 4: Supplementary Figure S1.** ShinyGO analysis: significantly enriched KEGG pathway LYSOSOME. The genes iB-02516, iB-05119, iB-09043, and iB-09239 encoding ACP2, CD63 (LIMP), FGE, and AP-3 respectively, are all involved in lysosome function (ko04142) [52].

**Additional file 5: Supplementary Figure S2.** BlastKOALA analysis: KEGG pathway MINERAL ABSORPTION. The genes iB-00105, iB-02517, and iB-09991 encoding Ferritin, ATP7A, and sodium/potassium-transporting ATPase subunit beta, respectively, are all involved in mineral absorption (ko04978) [52].

**Additional file 6: Supplementary Figure S3.** BRITE analyses of KEGG orthologies. The column charts represent the number of genes that have been assigned to the different KEGG orthology pathways in the Brite analyses of BlastKOALA (A) and eggNOG (B).

**Additional file 7: Supplementary Table S4.** Expression data of iBeetle-detected genes involved in stink gland function. To present the expression data of the 130 genes identified in the 1^st^ and 2^nd^ phase of the iBeetle screen, we have extracted read counts and the glandspecific fold change expression from the transcriptomics data published in 2013 [28]. The gene indicated in bold has been identified both by the phenoytpic iBeetle screen as well as the transcriptomics approach.

**Additional file 8: Supplementary Table S5.** Representation of transcriptomics-identified genes in the iBeetle screen. Phenotype descriptions of the transcriptomics analysis are taken from Li et al. [28], iBeetle-phenotypes unchanged form wild type are indicated with 'non detected' (n.d.). Genes that have not been analyzed for a stink gland phenotype as they were only part of the pupal screen of the first phase are listed as not analyzed (n.a.). The 13 genes causing a gland phenotype upon knock-down and were also covered in the iBeetle screen are marked in *italics*. RNAi-knockdown of genes highlighted in bold resulted in easily detectable and strikingly changed stink glands, and therefore should have been detected in the iBeetle screen. However, only one of the two (underlined) was detected. Changes in annotations between Tcas3.0 and Tcas5.2 are provided.

**Additional file 9: Supplementary Table S6.** Non-overlapping fragments (NOFs) for rescreen in 1st phase of iBeetle. In case there is partial overlap with the original iBeetle fragment, this is indicated. Sequence parts of primers used for amplification are underlined.

**Additional file 10: Supplementary Table S7.** Non-overlapping fragments (NOFs) for rescreen in 2nd phase of iBeetle. The NOFs were ordered ready to use from Eupheria Biotech GmbH (Dresden, Germany), which also determined the best sequence.

**Additional file 11.** FASTA file 69 proteins (separate text file). Amino acid sequences in FASTA format of the 69 encoded proteins used for analyzes in BlastKOALA and eggNOGmapper with the identifiers listed as ‘query’ in Additional file 2: Supplementary Table S2.

## Data Availability

The datasets of the iBeetle screen including the fragments used as double-strand RNAs for the gene knock-downs are available in iBeetle-Base (https://ibeetle-base.uni-goettingen.de/) [38; 39]. All other data analysed and generated during this study are cited, or included in this published article and its supplementary information files, respectively.

## Abbreviations

1-C15: 1-Pentadecene
1-C17: 1-Heptadecene
conf: confirmed
dsRNA: Double-stranded RNA
EBQ: 2-Ethyl-1,4-benzoquinone
FC: Fold change
GC–MS: Gas chromatography-mass spectrometry
MBQ: 2-Methyl-1,4-benzoquinone
n.a.: not analyzed
n.d.: non detected
NOFs: Non-overlapping fragments
OGS: Official gene set
RNAi: RNA interference
RNA-Seq: Next generation sequencing of mRNA
Tcas3.0: Official assembly of genomic sequence of *Tribolium castaneum* version 3 0
Tcas5.2: Official assembly of genomic sequence of *Tribolium castaneum* version 5.2
