# Supplementary material for: Phenotypic screen and transcriptomics approach complement each other in functional genomics of defensive stink gland physiology
Source: BMC Genomics. 2022 Aug 20;23:608. doi: 10.1186/s12864-022-08822-z (PMC9392906; doi:10.1186/s12864-022-08822-z)
Supplement: Supplementary file 1 — Additional file 1: Supplementary Table S1. iBeetle-identified genes involved in defensive stink gland function. The phenotype recognized in the first-pass iBeetle screen and annotated in iBeetle-Base (original detection in 1st or 2nd phase indicated) is compared to the categorized (Fig. 1) phenoytpes of the re-secreen, with phenotypes unchanged form wild type declared as ‘non detected’ (n.d.). The 60 re-screen-confirmed gland phenotypes are indicated in bold. Changes in annotations between Tcas3.0 and Tcas5.2 are provided in brackets. Gland-specific higher expression (FC ≥ 2; Additional file 3: Table S3) is indicted by an “x”. [file 12864_2022_8822_MOESM1_ESM.pdf]

Additional file 1: Table S1 iBeetle-identified genes involved in defensive stink gland function

iBeetle			re-screen categorized phenotype	OGS_# (ass. 5.2)	FC ≥ 2
iB_#	phase	phenotype			
**iB_00081**	**1st**	**abd gland partially color darker**	**irregular separation**	**Tc_000379**	**x**
**iB_00105**	**2nd**	**both glands darker, abd content decreased**	**empty/necrotic**	**Tc_000476**	
**iB_00110**	**1st**	**abd gland size increased**	**irregular reservoir size**	**Tc_000504**	**x**
**iB_00185**	**1st**	**abd color not present and shape irregular**	**empty/necrotic**	**Tc_000885**	
**iB_00414**	**1st**	**prothoracic glands partially color darker**	**secretion color darker**	**Tc_002616**	
iB_00754	1st	abd partially color darker	n.d.	Tc_031464 (Tc_004698)	
**iB_01044**	**1st**	**abd color darker**	**irregular separation**	**Tc_006408**	
iB_01236	1st	abd gland size decreased and irregular shape	n.d.	Tc_007650	
**iB_01372**	**1st**	**abd color darker**	**irregular separation**	**Tc_033883 (Tc_008608)**	
iB_01440	1st	abd gland color not present	n.d.	Tc_008936	
iB_01644	1st	abd color darker	n.d.	Tc_010033	
**iB_01798**	**1st**	**abd glands sometimes darker**	**irregular separation**	**Tc_011075**	
iB_01814	1st	abd glands mostly color darker	n.d.	Tc_011159	
iB_01910	1st	abd glands slightly color darker	n.d.	Tc_011969	
**iB_01975**	**2nd**	**both glands darker, abd content decreased**	**secretion color darker**	**Tc_012387**	
**iB_02292**	**1st**	**abd size decreased and partially color not present**	**less secretion**	**Tc_014494**	
**iB_02297**	**1st**	**unspecified alteration**	**empty/necrotic**	**Tc_014520**	
iB_02301	1st	abd color darker	n.d.	Tc_014544	
iB_02367	1st	abd gland color darker	n.d.	Tc_014967	
**iB_02401**	**1st**	**abd gland color darker**	**secretion color darker**	**Tc_015095**	
**iB_02416**	**1st**	**abd gland partially slightly color darker**	**irregular separation**	**Tc_015165**	**x**
**iB_02428**	**1st**	**abd gland shape and content irregular**	**irregular separation**	**Tc_015203**	
**iB_02471**	**1st**	**prothoracic glands partially darker color, abd glands size decreased**	**irregular reservoir size**	**Tc_015379**	**x**
**iB_02516**	**1st**	**glands content darker**	**melanized**	**Tc_015811**	**x**
**iB_02517**	**1st**	**partially content darker**	**melanized**	**Tc_030914 (Tc_015817, Tc_015818)**	
iB_02542	1st	gland content partially darker	n.d.	Tc_015993	
**iB_02563**	**1st**	**partially content not present**	**empty/necrotic**	**Tc_030950 (Tc_016253, Tc_016254)**	
iB_02584	1st	gland content not present	n.d.	Tc_030051	
iB_02625	1st	gland shape irregular	n.d.	Tc_011255	
**iB_02627**	**1st**	**abd glands partially content not present**	**less secretion**	**Tc_011288**	
iB_02633	1st	gland shape irregular, content partially not present	n.d.	Tc_031391 (Tc_011371)	
**iB_02673**	**1st**	**irregular shape, content partially not present**	**empty/necrotic**	**Tc_032251 (Tc_000239, Tc_000240)**	
iB_02692	1st	abd glands partially content not present	n.d.	Tc_003063	
**iB_02716**	**1st**	**abd glands mostly content not present, gland shape irregular**	**empty/necrotic**	**Tc_031046 and Tc_032493 (Tc_002723)**	
iB_02743	2nd	abd gland content partially not present	n.d.	Tc_003968	
**iB_02774**	**2nd**	**abd gland content darker**	**secretion color darker**	**Tc_008303**	
**iB_02931**	**1st**	**abd glands content darker**	**irregular separation**	**Tc_031247 (Tc_011810, Tc_011812)**	**x**
**iB_03294**	**2nd**	**abd gland content darker**	**secretion color darker**	**Tc_002074**	
iB_03401	1st	glands partially color darker	n.d.	Tc_002550	
**iB_03552**	**1st**	**gland color darker and partially decreased size**	**secretion color darker**	**Tc_032367 (Tc_003409)**	**x**
iB_03637	1st	sometimes gland size increased	n.d.	Tc_003857	

Additional file 1: Table S1 iBeetle-identified genes involved in defensive stink gland function

iB_03693	1st	gland size increased	n.d.	Tc_031485 (Tc_004126)	
**iB_03695**	**1st**	**gland color darker**	**irregular separation**	**Tc_004129**	
iB_03780	1st	glands darker	n.d.	Tc_031955 (Tc_004533, Tc_004534)	x
**iB_03913**	**1st**	**abd gland structure necrotic**	**empty/necrotic**	**Tc_005167**	
iB_04066	1st	abd gland color not present	n.d.	Tc_033952 (Tc_006098)	
iB_04137	1st	abd gland size increased	n.d.	Tc_034081 (Tc_006423)	
iB_04205	1st	abd glands not present	n.d.	Tc_006735	
iB_04420	2nd	abd mostly content decreased	irregular separation	Tc_008047	
**iB_04702**	**2nd**	**prothoracic glands darker**	**secretion color darker**	**Tc_031200 (Tc_009790, Tc_009792)**	
**iB_04717**	**2nd**	**both glands darker and content fragmented**	**melanized**	**Tc_009877**	
**iB_04797**	**1st**	**abd gland size decreased and lighter color**	**colorless**	**Tc_010251**	**x**
iB_04839	1st	both glands darker	n.d.	Tc_010449	
iB_04850	1st	abd glands partially color darker	n.d.	Tc_010484	
iB_05119	2nd	both glands darker, abd gland size decreased	less secretion	Tc_034419 (Tc_011865)	x
**iB_05264**	**1st**	**gland content mostly not present**	**empty/necrotic**	**Tc_012539**	
**iB_05278**	**1st**	**abd glands mostly content not present**	**empty/necrotic**	**Tc_034399 (Tc_012607, Tc_012609, Tc_012610)**	
iB_05284	1st	abd glands content slightly darker	n.d.	Tc_034421 (Tc_012641, Tc_012642, Tc_012643, Tc_012644)	
**iB_05329**	**1st**	**abd glands content darker**	**irregular separation**	**Tc_012828**	
**iB_05331**	**1st**	**abd glands partially content not present**	**less secretion**	**Tc_012834**	
iB_05342	1st	abd glands content darker	n.d.	Tc_012857	
**iB_05442**	**1st**	**unspecified alteration**	**colorless**	**Tc_033122 (Tc_013511, Tc_013513)**	**x**
**iB_05518**	**1st**	**abd gland color darker**	**melanized**	**Tc_032992 (Tc_013892)**	
iB_05584	1st	abd glands partially color darker	n.d.	Tc_014205	
**iB_05712**	**1st**	**abd glands mostly content not present**	**less secretion**	**Tc_033471 (Tc_014869, Tc_014870)**	
**iB_05719**	**1st**	**abd glands partially content darker**	**irregular separation**	**Tc_014887**	
iB_05874	2nd	prothoracic glands darker	n.d.	Tc_015692	
iB_05942	2nd	abd gland size decreased, content darker	n.d.	Tc_031853 (Tc_016013, Tc_016016, Tc_016017, Tc_016018)	
iB_06333	2nd	abd gland content not present	n.d.	Tc_014550	
**iB_06359**	**2nd**	**abd content decreased and partially darker**	**secretion color darker**	**Tc_031191 (Tc_015926, Tc_015905)**	
iB_06684	2nd	abd content and size decreased, and abd content darker	n.d.	Tc_034492 (Tc_001853, Tc_001855)	
iB_06779	2nd	prothoracic glands darker	n.d.	Tc_004179	

Additional file 1: Table S1 iBeetle-identified genes involved in defensive stink gland function

**iB_06806**	**2nd**	**glands color darker and content fragmented**	**melanized**	**Tc_000393**	
iB_06868	2nd	abd gland color darker	n.d.	Tc_003208	
iB_07043	2nd	"stink glands"	less secretion	Tc_003827	
iB_07188	2nd	abd gland size increased, content opaque	n.d.	Tc_032832<br>(Tc_008176)	
**iB_07361**	**2nd**	**prothoracic glands darker**	**melanized**	**Tc_009459**	
iB_07747	2nd	abd content lighter and fragmented	n.d.	Tc_031768<br>(Tc_010623,<br>Tc_010625)	
iB_07759	2nd	abd gland size and content decreased	n.d.	Tc_030320	
iB_07760	2nd	abd content decreased	n.d.	Tc_031822<br>(Tc_016034)	
iB_07764	2nd	abd content lighter & fragmented	n.d.	Tc_005075	
iB_07772	2nd	abd content decreased	n.d.	Tc_011144	
iB_07782	2nd	abd gland content spotted and partially darker	n.d.	Tc_006837	
iB_07783	2nd	abd gland content fragmented	n.d.	Tc_031904<br>(Tc_002154,<br>Tc_002155)	
iB_07900	2nd	abd content lighter	secretion color darker	Tc_001243	
iB_07902	2nd	abd content decreased, fragmented	secretion color darker	Tc_001275	
iB_07917	2nd	abd content decreased, fragmented	n.d.	Tc_000183	
**iB_07918**	**2nd**	**abd content fragmented or potentially not present**	**less secretion**	**Tc_001376**	**x**
iB_07926	2nd	abd content split, lighter color, and size decreased	n.d.	Tc_003231	
iB_08184	2nd	abd gland color darker	less secretion	Tc_032964	
iB_08303	2nd	abd content potentially not present or darker	n.d.	Tc_030655	
**iB_08398**	**2nd**	**gland color darker,<br>abd content partially not present**	**secretion color darker**	**Tc_033206**	
iB_08468	2nd	abd gland content not present	secretion color darker	Tc_015328	
**iB_08506**	**2nd**	**abd gland size and content decreased**	**less secretion**	**Tc_015537**	
iB_08561	2nd	abd gland size and content decreased	n.d.	Tc_004632	x
iB_08587	2nd	abd gland content not present, size decreased	n.d.	Tc_009420	
iB_08666	2nd	abd size decreased, and content split	n.d.	Tc_009201	
**iB_08760**	**2nd**	**abd gland content darker**	**melanized**	**Tc_033755<br>(Tc_009924)**	
**iB_08861**	**2nd**	**abd gland color decreased**	**melanized**	**Tc_006177**	
**iB_09043**	**2nd**	**both glands color darker,<br>abd gland color not present**	**colorless**	**Tc_016314**	**x**
**iB_09050**	**2nd**	**both glands color darker,<br>abd gland content decreased**	**less secretion**	**Tc_033022<br>(Tc_013879,<br>Tc_013880)**	
**iB_09103**	**2nd**	**abd content darker**	**melanized**	**Tc_014482**	
**iB_09239**	**2nd**	**abd gland content and size decreased**	**less secretion**	**Tc_008912**	
iB_09272	2nd	both gland content color darker,<br>abd gland content not present	n.d.	Tc_006097	
iB_09308	2nd	abd content decreased, not present,<br>or partially darker	n.d.	Tc_005489	
iB_09311	2nd	both glands color darker	n.d.	Tc_006635	
iB_09326	2nd	abd gland content decreased, irregular shape	n.d.	Tc_030081	
iB_09329	2nd	abd gland content not present,<br>glands partially not present	n.d.	Tc_030130	
iB_09337	2nd	both glands color darker	n.d.	Tc_030243	
iB_09340	2nd	abd gland content not present, or darker	n.d.	Tc_032272<br>(Tc_000166,<br>Tc_030316)	
**iB_09355**	**2nd**	**both glands color darker**	**secretion color darker**	**Tc_006363**	
iB_09403	2nd	abd gland content fragmented,<br>abd gland color darker	n.d.	Tc_034170	
**iB_09413**	**2nd**	**both glands color darker**	**melanized**	**Tc_005389**	**x**
**iB_09430**	**2nd**	**both glands darker and fragmented**	**secretion color darker**	**Tc_005306**	

iB_09661	2nd	abd glands potentially content decreased	n.d.	Tc_001099	x
iB_09736	2nd	abd gland color decreased	secretion color darker	Tc_003116	
iB_09896	2nd	abd gland content darker	n.d.	Tc_007557	
iB_09910	2nd	abd gland content decreased	n.d.	Tc_007292	
iB_09924	2nd	abd gland content potentially darker	n.d.	Tc_008270	
**iB_09988**	**2nd**	**abd gland content darker and decreased**	**less secretion**	**Tc_014025**	
**iB_09991**	**2nd**	**abd gland color darker**	**secretion color darker**	**Tc_014033**	
iB_10007	2nd	abd gland content split	n.d.	Tc_033075	
**iB_10104**	**2nd**	**both glands color darker**	**secretion color darker**	**Tc_013627**	
**iB_10133**	**2nd**	**abd gland content potentially not present or decreased**	**less secretion**	**Tc_014774**	
**iB_10156**	**2nd**	**abd gland content darker, or content not present**	**less secretion**	**Tc_033320 (Tc_015307)**	
iB_10159	2nd	abd gland content decreased or not present	secretion color darker	Tc_015429	x
**iB_10181**	**2nd**	**both glands color darker**	**secretion color darker**	**Tc_015547**	
iB_10206	2nd	abd gland content decreased or not present	n.d.	Tc_015049	
**iB_10701**	**2nd**	**abd gland content darker and decreased**	**melanized**	**Tc_014985**	
iB_10748	2nd	abd gland content not present	n.d.	Tc_007828	

n.d. non detected (= wildtype)
bold confirmed specific gland phenotype
() former gene annotation (ass. 3.0)
