# Supplementary material for: Phenotypic screen and transcriptomics approach complement each other in functional genomics of defensive stink gland physiology
Source: BMC Genomics. 2022 Aug 20;23:608. doi: 10.1186/s12864-022-08822-z (PMC9392906; doi:10.1186/s12864-022-08822-z)
Supplement: Supplementary file 4 — Additional file 4: Supplementary Figure S1. ShinyGO analysis: significantly enriched KEGG pathway LYSOSOME. The genes iB-02516, iB-05119, iB-09043, and iB-09239 encoding ACP2, CD63 (LIMP), FGE, and AP-3 respectively, are all involved in lysosome function (ko04142) [52]. [file 12864_2022_8822_MOESM4_ESM.pdf]

Additional file 4: Supplementary Figure S1:

ShinyGO analysis: significant KEGG pathway LYSOSOME

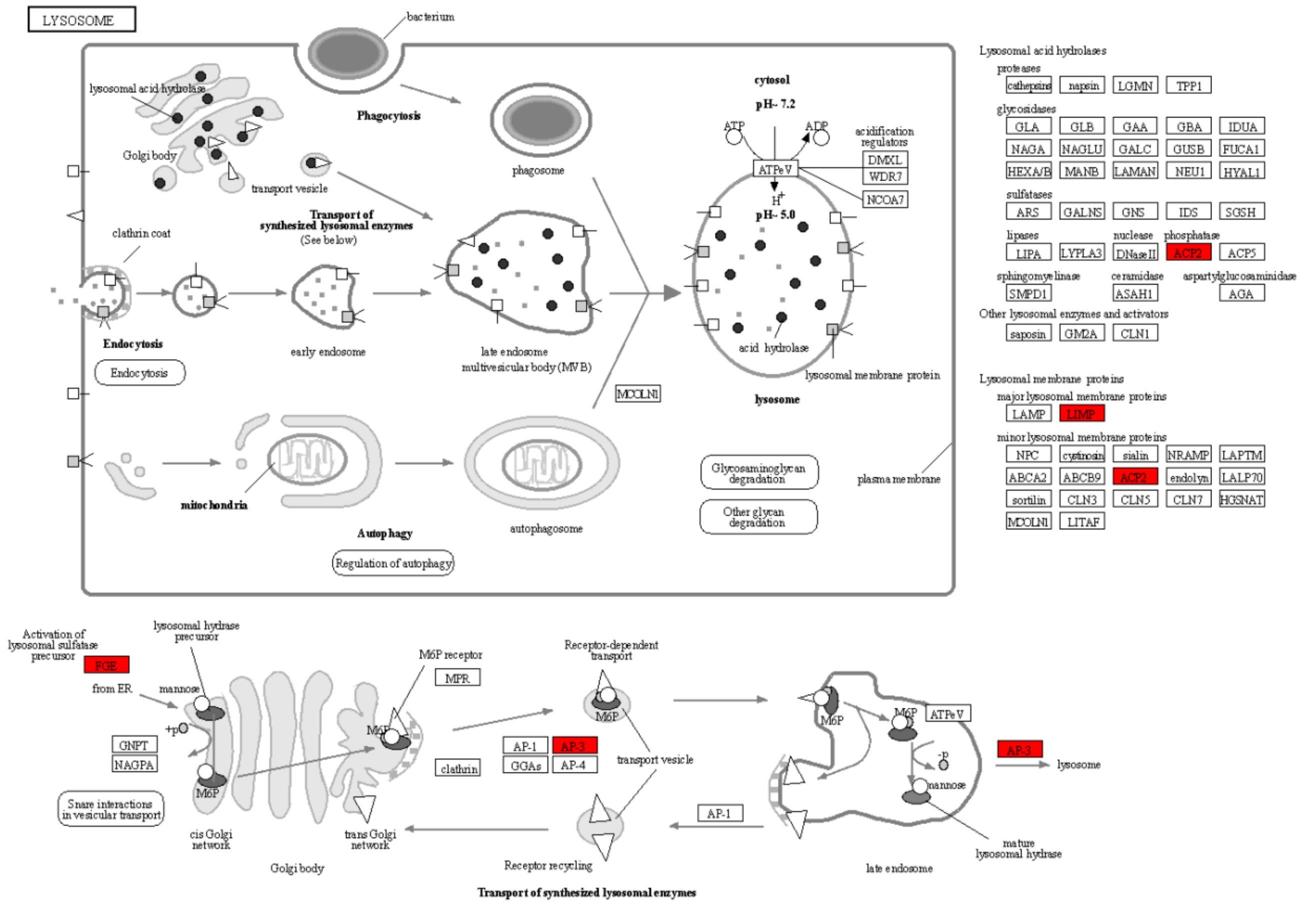

Data on KEGG graph
Rendered by Pathview
