# Supplementary material for: Phenotypic screen and transcriptomics approach complement each other in functional genomics of defensive stink gland physiology
Source: BMC Genomics. 2022 Aug 20;23:608. doi: 10.1186/s12864-022-08822-z (PMC9392906; doi:10.1186/s12864-022-08822-z)

Additional file 5: Supplementary Figure S2:
BlastKOALA analysis: KEGG pathway MINERAL ABSORPTION

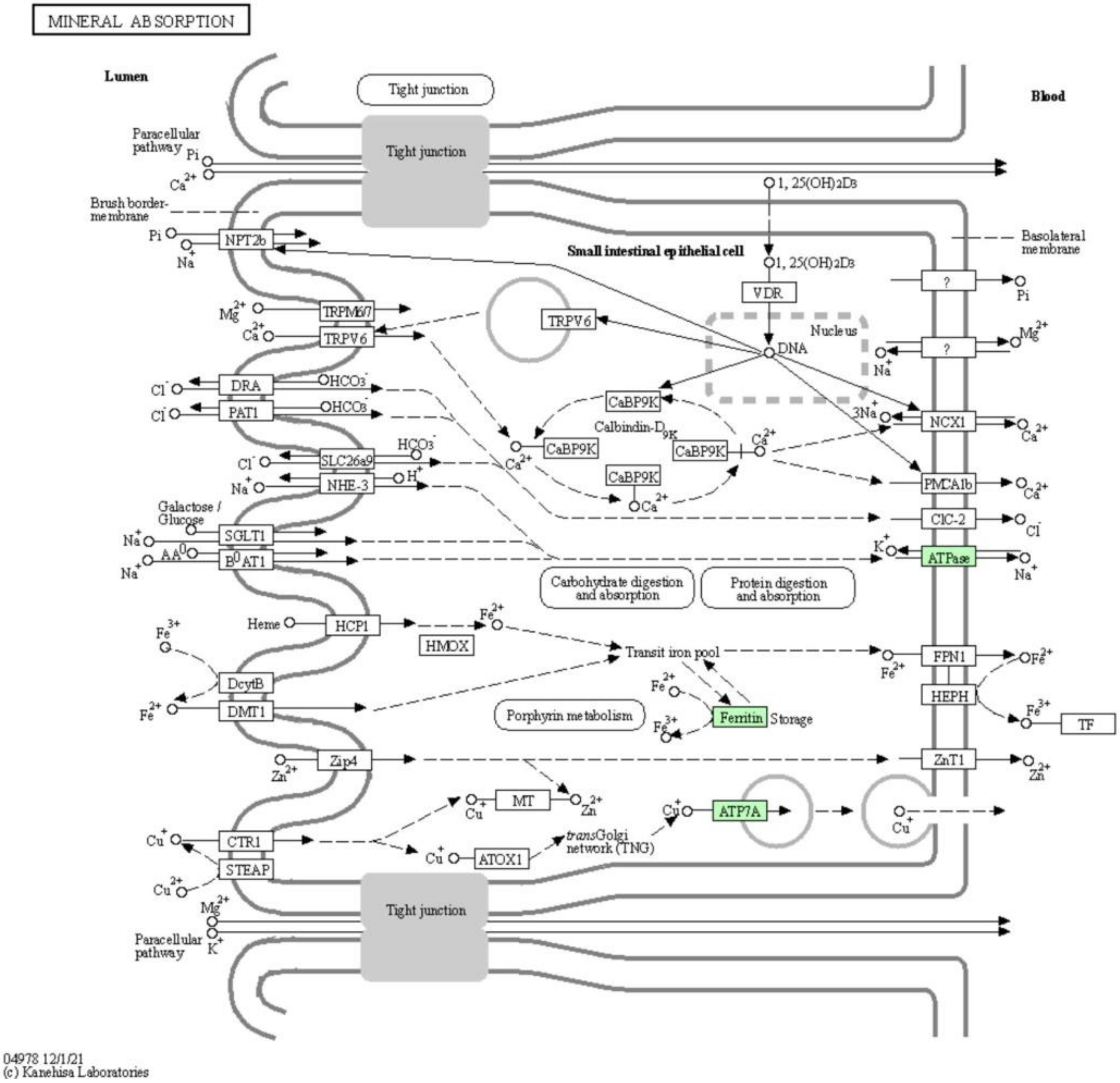

Supplement: Supplementary file 5 — Additional file 5: Supplementary Figure S2. BlastKOALA analysis: KEGG pathway MINERAL ABSORPTION. The genes iB-00105, iB-02517, and iB-09991 encoding Ferritin, ATP7A, and sodium/potassium-transporting ATPase subunit beta, respectively, are all involved in mineral absorption (ko04978) [52]. [file 12864_2022_8822_MOESM5_ESM.pdf]
