# Supplementary figures and images for: Phenotypic screen and transcriptomics approach complement each other in functional genomics of defensive stink gland physiology
Source: BMC Genomics. 2022 Aug 20;23:608. doi: 10.1186/s12864-022-08822-z (PMC9392906; doi:10.1186/s12864-022-08822-z)

**A** BlastKOALA: BRITE analysis of KEGG orthologies

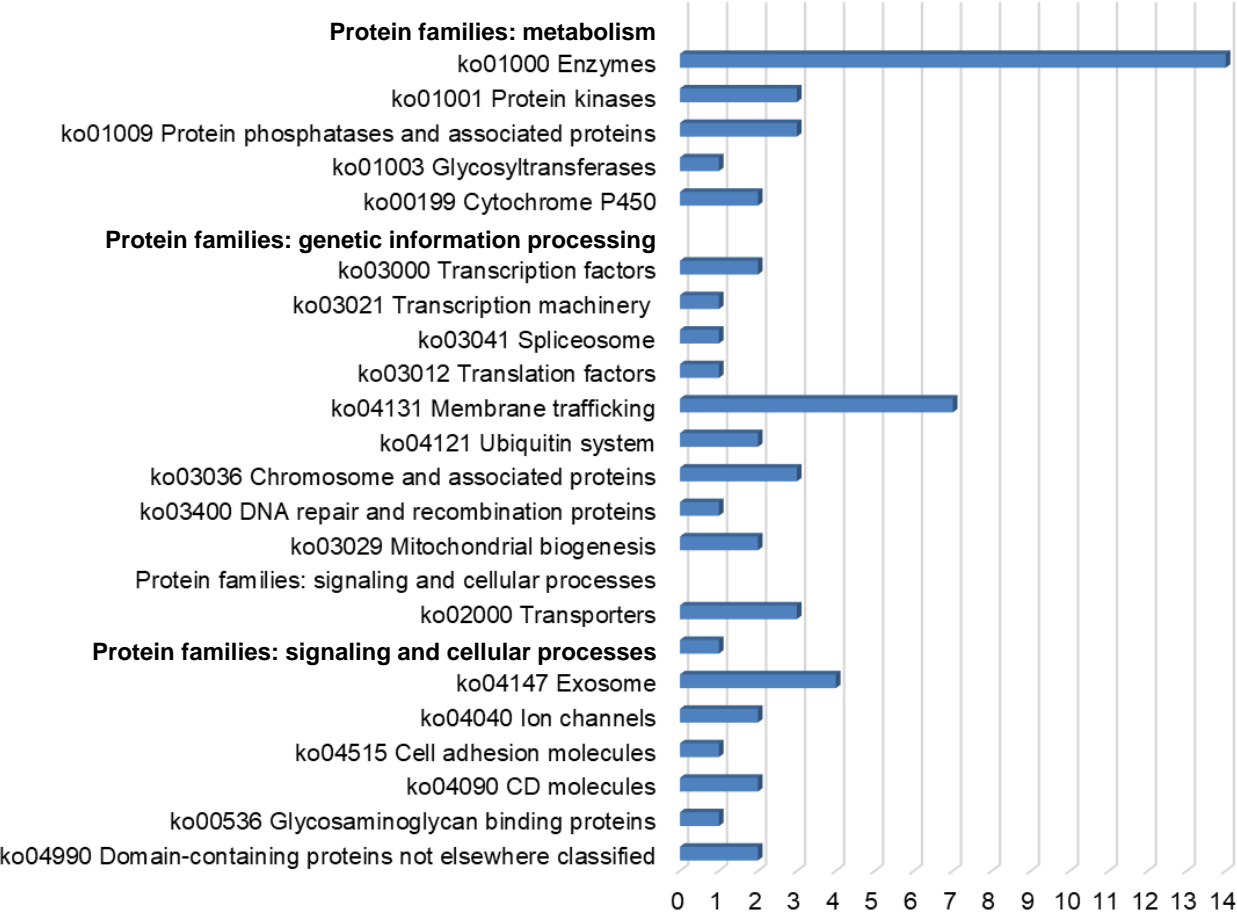

**B** eggNOG: BRITE analysis of KEGG orthologies

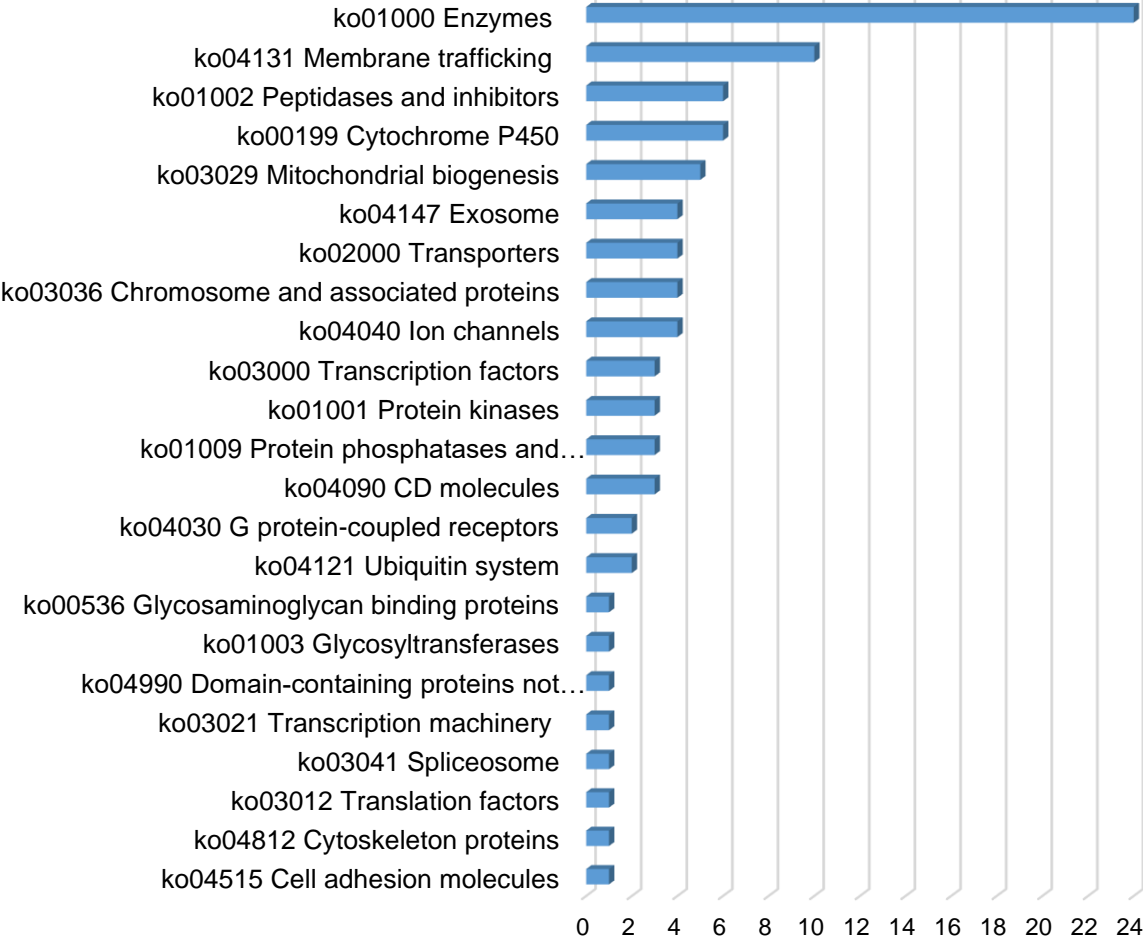

Supplement: Supplementary file 6 — Additional file 6: Supplementary Figure S3. BRITE analyses of KEGG orthologies. The column charts represent the number of genes that have been assigned to the different KEGG orthology pathways in the Brite analyses of BlastKOALA (A) and eggNOG (B). [file 12864_2022_8822_MOESM6_ESM.pdf]
