# Supplementary material for: Phenotypic screen and transcriptomics approach complement each other in functional genomics of defensive stink gland physiology
Source: BMC Genomics. 2022 Aug 20;23:608. doi: 10.1186/s12864-022-08822-z (PMC9392906; doi:10.1186/s12864-022-08822-z)
Supplement: Supplementary file 7 — Additional file 7: Supplementary Table S4. Expression data of iBeetle-detected genes involved in stink gland function. To present the expression data of the 130 genes identified in the 1st and 2nd phase of the iBeetle screen, we have extracted read counts and the glandspecific fold change expression from the transcriptomics data published in 2013 [28]. The gene indicated in bold has been identified both by the phenoytpic iBeetle screen as well as the transcriptomics approach. [file 12864_2022_8822_MOESM7_ESM.pdf]

Additional file 7: Table S4 **Expression data of iBeetle-detected genes involved in stink gland function**

iB_#	OGS_# (ass.3.0)	male - thx		female - thx		male - abd		female - abd		reference tissue (mid abdomen)		log2 FC (sample reads/reference reads)			
		reads	depth	reads	depth	reads	depth	reads	depth	reads	depth	male - thx	female - thx	male - abd	female - abd
iB_00081	Tc_000379	2466	143,28	2331	135,44	2332	135,50	3090	179,54	1031	59,91	1,258	1,177	1,178	1,584
iB_00105	Tc_000476	29304	1718,44	30781	1805,06	27672	1622,74	17243	1011,16	17513	1027,00	0,743	0,814	0,660	-0,022
iB_00110	Tc_000504	45	0,92	99	2,02	85	1,73	122	2,49	13	0,27	1,791	2,929	2,709	3,230
iB_00185	Tc_000885	144	5,85	113	4,59	54	2,19	10	0,41	157	6,37	-0,125	-0,474	-1,540	-3,973
iB_00414	Tc_002616	2227	58,77	2140	56,47	2323	61,30	3540	93,42	1747	46,10	0,350	0,293	0,411	1,019
iB_00754	Tc_004698	779	7,93	591	6,02	820	8,35	1094	11,14	2216	22,56	-1,508	-1,907	-1,434	-1,018
iB_01044	Tc_006408	7507	270,91	7545	272,28	8356	301,55	7917	285,70	14097	508,72	-0,909	-0,902	-0,755	-0,832
iB_01236	Tc_007650	361	6,30	356	6,21	423	7,38	450	7,85	336	5,86	0,104	0,083	0,332	0,421
iB_01372	Tc_008608	144	1,72	69	0,83	179	2,14	176	2,11	2111	25,25	-3,874	-4,935	-3,560	-3,584
iB_01440	Tc_008936	162	6,68	170	7,01	174	7,18	140	5,78	1216	50,17	-2,908	-2,839	-2,805	-3,119
iB_01644	Tc_010033	926	39,76	978	41,99	1311	56,29	871	37,40	603	25,89	0,619	0,698	1,120	0,531
iB_01798	Tc_011075	153	10,77	105	7,39	150	10,56	625	43,98	3516	247,42	-4,522	-5,065	-4,551	-2,492
iB_01814	Tc_011159	1588	55,72	1479	51,89	956	33,54	847	29,72	1829	64,18	-0,204	-0,306	-0,936	-1,111
iB_01910	Tc_011969	3	0,09	0	0	587	16,82	2	0,06	3	0,09	0	-	7,612	-0,585
iB_01975	Tc_012387	2	0,03	0	0	2716	34,13	0	0	4	0,05	-1,000	-	9,407	-
iB_02292	Tc_014494	456	13,25	492	14,29	546	15,86	463	13,45	372	10,81	0,294	0,403	0,554	0,316
iB_02297	Tc_014520	556	11,40	499	10,23	576	11,81	521	10,68	1025	21,01	-0,882	-1,039	-0,831	-0,976
iB_02301	Tc_014544	7	0,22	20	0,64	231	7,35	33	1,05	65	2,07	-3,215	-1,700	1,829	-0,978
iB_02367	Tc_014967	115	3,71	106	3,42	140	4,51	116	3,74	78	2,51	0,560	0,443	0,844	0,573
iB_02401	Tc_015095	1527	39,15	1831	46,95	1367	35,05	1031	26,44	881	22,59	0,793	1,055	0,634	0,227
iB_02416	Tc_015165	550	30,42	515	28,49	557	30,81	425	23,51	241	13,33	1,190	1,096	1,209	0,818
iB_02428	Tc_015203	427	10,36	344	8,35	515	12,50	73	1,77	251	6,09	0,767	0,455	1,037	-1,782
iB_02471	Tc_015379	616	16,57	487	13,10	2226	59,86	3212	86,38	132	3,55	2,222	1,883	4,076	4,605
iB_02516	Tc_015811	682	21,23	757	23,56	881	27,42	814	25,33	367	11,42	0,894	1,045	1,263	1,149
iB_02517	Tc_015817	4	0,44	3	0,33	11	1,20	5	0,55	31	3,39	-2,954	-3,369	-1,495	-2,632
	Tc_015818	1563	16,68	1182	12,61	2019	21,55	1442	15,39	1537	16,40	0,024	-0,379	0,394	-0,092
iB_02542	Tc_015993	6654	170,62	5142	131,85	1761	45,15	1126	28,87	4431	113,62	0,587	0,215	-1,331	-1,976
iB_02563	Tc_016253	50	2,48	65	3,23	52	2,58	41	2,04	234	11,62	-2,227	-1,848	-2,170	-2,513
	Tc_016254	767	10,69	710	9,89	3365	46,89	10312	143,69	2551	35,55	-1,734	-1,845	0,400	2,015
iB_02584	Tc_030051	1210	29,08	1089	26,17	1279	30,74	1205	28,96	621	14,93	0,962	0,810	1,042	0,956
iB_02625	Tc_011255	466	5,26	421	4,75	836	9,43	757	8,54	1731	19,52	-1,893	-2,040	-1,050	-1,193
iB_02627	Tc_011288	156	1,66	115	1,23	95	1,01	60	0,64	1637	17,44	-3,391	-3,831	-4,107	-4,770
iB_02633	Tc_011371	275	4,58	222	3,70	265	4,41	285	4,74	774	12,88	-1,493	-1,802	-1,546	-1,441
iB_02673	Tc_000239	1	0,19	1	0,19	1	0,19	1	0,19	10	1,86	-3,322	-3,322	-3,322	-3,322
	Tc_000240	16	0,40	22	0,55	16	0,40	36	0,90	279	6,97	-4,124	-3,665	-4,124	-2,954
iB_02692	Tc_003063	489	2,50	363	1,86	913	4,67	1518	7,77	3242	16,59	-2,729	-3,159	-1,828	-1,095
iB_02716	Tc_002723	2823	12,35	2279	9,97	3947	17,27	6931	30,33	4815	21,07	-0,770	-1,079	-0,287	0,526
iB_02743	Tc_003968	29	1,48	19	0,97	99	5,04	58	2,95	56	2,85	-0,949	-1,559	0,822	0,051
iB_02774	Tc_008303	419	6,28	343	5,14	448	6,72	449	6,73	620	9,29	-0,565	-0,854	-0,469	-0,466
iB_02931	Tc_011810	660	76,70	547	63,57	698	81,11	148	17,20	136	15,80	2,279	2,008	2,360	0,122
	Tc_011812	7031	261,94	7559	281,61	8061	300,31	2731	101,74	951	35,43	2,886	2,991	3,083	1,522
iB_03294	Tc_002074	347	4,33	314	3,92	384	4,79	340	4,24	806	10,06	-1,216	-1,360	-1,070	-1,245
iB_03401	Tc_002550	607	13,05	503	10,82	699	15,03	637	13,70	668	14,37	-0,138	-0,409	0,065	-0,069
iB_03552	Tc_003409	44	1,86	49	2,07	40	1,69	53	2,24	19	0,80	1,212	1,367	1,074	1,480
iB_03637	Tc_003857	0	0	0	0	2	0,07	0	0	45	1,50	-	-	-4,492	-
iB_03693	Tc_004126	91	0,56	41	0,25	25	0,15	17	0,10	821	5,02	-3,173	-4,324	-5,037	-5,594
iB_03695	Tc_004129	8	0,18	6	0,13	8	0,18	0	0	41	0,92	-2,358	-2,773	-2,358	-
iB_03780	Tc_004533	370	19,21	357	18,53	364	18,90	470	24,40	249	12,93	0,571	0,520	0,548	0,917
	Tc_004534	847	34,28	783	31,69	903	36,54	876	35,4505	384	15,5399	1,141	1,028	1,234	1,190
iB_03913	Tc_005167	670	9,19	672	9,22	1101	15,11	1069	14,67	1211	16,62	-0,854	-0,850	-0,137	-0,180
iB_04066	Tc_006098	12	0,07	16	0,09	40	0,23	36	0,21	2391	13,94	-7,638	-7,223	-5,901	-6,053
iB_04137	Tc_006423	2328	35,79	2109	32,42	1977	30,39	1778	27,33	1746	26,84	0,415	0,273	0,179	0,026
iB_04205	Tc_006735	248	4,47	232	4,19	3447	62,20	351	6,33	874	15,77	-1,817	-1,914	1,980	-1,316
iB_04420	Tc_008047	0	0	0	0	0	0	0	0	0	0	-	-	-	-
iB_04702	Tc_009790	404	33,8896	360	30,20	368	30,8698	183	15,35	450	37,75	-0,156	-0,322	-0,290	-1,298
	Tc_009792	2016	60,37	1991	59,62	1705	51,06	1261	37,76	1457	43,63	0,468	0,450	0,227	-0,208
iB_04717	Tc_009877	3	0,07	4	0,09	82	1,92	20	0,47	26	0,61	-3,115	-2,700	1,657	-0,379
iB_04797	Tc_010251	29710	760,26	33432	855,50	39562	1012,36	14185	362,98	1510	38,64	4,298	4,469	4,711	3,232
iB_04839	Tc_010449	963	62,23	754	48,73	406	26,24	866	55,97	555	35,87	0,795	0,442	-0,451	0,642
iB_04850	Tc_010484	341	11,16	172	5,63	312	10,21	1354	44,32	3204	104,87	-3,232	-4,219	-3,360	-1,243
iB_05119	Tc_011865	8620	328,88	7351	280,46	11479	437,954	11409	435,283	3498	133,458	1,301	1,071	1,714	1,706
iB_05264	Tc_012539	245	1,60	190	1,24	280	1,82	403	2,63	984	6,41	-2,006	-2,373	-1,813	-1,288
iB_05278	Tc_012607	0	0	0	0	1	0,10	0	0	3	0,31	-	-	-1,585	-
	Tc_012609	8	0,21	5	0,13	8	0,21	105	2,78	7	0,19	0,193	-0,485	0,193	3,907
	Tc_012610	85	1,02	43	0,51	109	1,30	69	0,82	147	1,76	-0,790	-1,773	-0,431	-1,091
iB_05284	Tc_012641	10	0,56	6	0,34	41	2,31	16	0,90	1993	112,20	-7,639	-8,376	-5,603	-6,961
	Tc_012642	0	0	0	0	43	2,04	0	0	1	0,05	-	-	5,426	-
	Tc_012643	0	0	0	0	33	1,9	0	0	0	0	-	-	-	-
	Tc_012644	0	0	0	0	467	13,5	0	0	0	0	-	-	-	-
iB_05329	Tc_012828	89	4,32	44	2,14	1020	49,50	433	21,01	927	44,99	-3,381	-4,397	0,138	-1,098
iB_05331	Tc_012834	449	4,54	401	4,05	672	6,79	486	4,91	931	9,40	-1,052	-1,215	-0,470	-0,938
iB_05342	Tc_012857	629	15,59	466	11,55	860	21,32	746	18,49	713	17,67	-0,181	-0,614	0,270	0,065
iB_05442	Tc_013511	238	11,46	238	11,46	418	20,13	127	6,12	10	0,48	4,573	4,573	5,385	3,667
	Tc_013513	444	15,12	484	16,48	826	28,13	204	6,95	21	0,72	4,402	4,527	5,298	3,280
iB_05518	Tc_013892	601	25,55	502	21,34	683	29,03	1110	47,18	1139	48,41	-0,922	-1,182	-0,738	-0,037
iB_05584	Tc_014205	302	20,90	268	18,55	340	23,53	401	27,76	350	24,23	-0,213	-0,385	-0,042	0,196
iB_05712	Tc_014869	0	0	1	0,02	1	0,02	0	0	44	0,87	-	-5,459	-5,459	-
	Tc_014870	1	0,01	2	0,03	9	0,13	0	0	77	1,14	-6,267	-5,267	-3,097	-

Additional file 7: Table S4 Expression data of iBeetle-detected genes involved in stink gland function

iB_05719	Tc_014887	210	10,43	168	8,35	504	25,04	2334	115,94	437	21,71	-1,057	-1,379	0,206	2,417
iB_05874	Tc_015692	137	3,59	108	2,83	73	1,91	101	2,65	141	3,70	-0,042	-0,385	-0,950	-0,481
iB_05942	Tc_016013	0	0	2	0,31	0	0	1	0,15	7	1,07	-	-1,807	-	-2,807
	Tc_016016	3	0,09	3	0,09	5	0,15	3	0,09	19	0,59	-2,663	-2,663	-1,926	-2,663
	Tc_016017	2	0,15	6	0,46	2	0,15	9	0,69	20	1,53	-3,322	-1,737	-3,322	-1,152
	Tc_016018	51	1,61	53	1,67	38	1,20	107	3,37	124	3,91	-1,282	-1,226	-1,706	-0,213
iB_06333	Tc_014550	7	0,34	4	0,19	137	6,60	17	0,82	24	1,16	-1,778	-2,585	2,513	-0,498
iB_06359	Tc_015905	0	0	0	0	180	12	0	0	6	0,40	-	-	4,907	-
	Tc_015926	31	2,97	47	4,51	57	5,47	66	6,33	46	4,41	-0,569	0,031	0,309	0,521
iB_06684	Tc_001853	1	0,04	3	0,11	0	0	1	0,04	2	0,07	-1,000	0,585	-	-1,000
	Tc_001855	2	0,06	0	0	0	0	1	0,03	3	0,09	-0,585	-	-	-1,585
iB_06779	Tc_004179	19	2,97	20	3,13	20	3,13	24	3,75	18	2,81	0,078	0,152	0,152	0,415
iB_06806	Tc_000393	163	3,96	284	6,89	210	5,10	7112	172,58	1192	28,92	-2,870	-2,069	-2,505	2,577
iB_06868	Tc_003208	535	23,21	501	21,73	761	33,01	500	21,69	406	17,61	0,398	0,303	0,906	0,300
iB_07043	Tc_003827	234	5,59	238	5,69	248	5,93	324	7,74	185	4,42	0,339	0,363	0,423	0,808
iB_07188	Tc_008176	1	0,02	6	0,11	15	0,27	0	0	9	0,16	-3,170	-0,585	0,737	-
iB_07361	Tc_009459	1	0,11	0	0	38	4,01	1	0,11	4	0,42	-2,000	-	3,248	-2,000
iB_07747	Tc_010623	1	0,06	1	0,06	1	0,06	2	0,11	1	0,06	0	0	0	1
	Tc_010625	11	0,11	2	0,02	7	0,07	14	0,14	26	0,25	-1,241	-3,700	-1,893	-0,893
iB_07759	Tc_030320	0	0	0	0	0	0	0	0	0	0	-	-	-	-
iB_07760	Tc_016034	79	1,59	26	0,52	53	1,07	32	0,65	371	7,48	-2,231	-3,835	-2,807	-3,535
iB_07764	Tc_005075	0	0,00	0	0,00	8	0,15	6	0,11	6	0,11	-	-	0,415	0,000
iB_07772	Tc_011144	583	16,30	514	14,37	639	17,87	600	16,78	430	12,02	0,439	0,257	0,571	0,481
iB_07782	Tc_006837	84	4,84	75	4,32	64	3,68	51	2,94	114	6,56	-0,441	-0,604	-0,833	-1,160
iB_07783	Tc_002154	7	0,24	4	0,14	4	0,14	0	0	0	0	-	-	-	-
	Tc_002155	1	0,02	0	0	0	0	0	0	0	0	-	-	-	-
iB_07900	Tc_001243	161	3,47	150	3,23	197	4,24	126	2,71	134	2,89	0,265	0,163	0,556	-0,089
iB_07902	Tc_001275	0	0	0	0	0	0	0	0	0	0	-	-	-	-
iB_07917	Tc_000183	319	25,25	336	26,60	277	21,93	238	18,84	226	17,89	0,497	0,572	0,294	0,075
iB_07918	Tc_013776	703	21,20	724	21,83	521	15,71	444	13,39	179	5,40	1,974	2,016	1,541	1,311
iB_07926	Tc_003231	8	0,50	17	1,06	10	0,62	6	0,37	104	6,46	-3,700	-2,613	-3,379	-4,115
iB_08184	Tc_032964	newly annotated gene													
iB_08303	Tc_030655	407	27,57	345	23,37	484	32,78	282	19,10	361	24,45	0,173	-0,065	0,423	-0,356
iB_08398	Tc_033206	newly annotated gene													
iB_08468	Tc_015328	1526	124,71	1615	131,98	2087	170,55	1157	94,55	1006	82,21	0,601	0,683	1,053	0,202
iB_08506	Tc_015537	740	40,93	570	31,53	816	45,14	528	29,21	461	25,50	0,683	0,306	0,824	0,196
iB_08561	Tc_004632	367	33,44	255	23,24	141	12,85	781	71,17	6	0,55	5,935	5,409	4,555	7,024
iB_08587	Tc_009420	462	4,19	455	4,13	667	6,05	1071	9,71	764	6,93	-0,726	-0,748	-0,196	0,487
iB_08666	Tc_009201	302	2,49	180	1,49	303	2,50	314	2,59	438	3,61	-0,536	-1,283	-0,532	-0,480
iB_08760	Tc_009422	781	48,97	714	44,77	819	51,36	518	32,48	415	26,02	0,912	0,783	0,981	0,320
iB_08861	Tc_006177	0	0	0	0	0	0	1	0,08	2	0,16	-	-	-	-1,000
iB_09043	Tc_016314	665	28,75	623	26,93	1315	56,85	1283	55,47	322	13,92	1,046	0,952	2,030	1,994
iB_09050	Tc_013879	159	5,19	119	3,88	145	4,73	120	3,92	210	6,86	-0,401	-0,819	-0,534	-0,807
	Tc_013880	0	0	0	0	0	0	0	0	1	0,20	-	-	-	-
iB_09103	Tc_014482	4	0,25	2	0,12	90	5,56	0	0	5	0,31	-0,322	-1,322	4,170	-
iB_09239	Tc_008912	257	16,95	207	13,66	170	11,22	203	13,39	294	19,40	-0,194	-0,506	-0,790	-0,534
iB_09272	Tc_006097	57	2,28	63	2,53	36	1,44	43	1,72	34	1,36	0,745	0,890	0,082	0,339
iB_09308	Tc_005489	12	0,44	12	0,44	4	0,15	1	0,04	11	0,41	0,126	0,126	-1,459	-3,459
iB_09311	Tc_006635	0	0	0	0	1	0,05	0	0	8	0,37	-	-	-3,000	-
iB_09326	Tc_030081	1	0,07	0	0	0	0	3	0,20	1	0,07	0,000	-	-	1,585
iB_09329	Tc_030130	0	0	0	0	0	0	0	0	0	0	-	-	-	-
iB_09337	Tc_030243	1	0,03	0	0	1	0,03	8	0,25	4	0,13	-2,000	-	-2,000	1,000
iB_09340	Tc_000166	4	0,13	18	0,6	7	0,23	0	0	1	0,03	2,000	4,170	2,807	-
	Tc_030316	2	0,07	2	0,07	3	0,10	0	0	0	0	-	-	-	-
iB_09355	Tc_006363	161	8,22	126	6,44	119	6,08	159	8,12	97	4,95	0,731	0,377	0,295	0,713
iB_09403	Tc_034170	newly annotated gene													
**iB_09413**	**Tc_005389**	**4693**	**97,77**	**4731**	**98,56**	**5706**	**118,88**	**1632**	**34,00**	**25**	**0,52**	**7,552**	**7,564**	**7,834**	**6,029**
iB_09430	Tc_005306	1414	34,51	1313	32,04	1591	38,83	1202	29,34	1226	29,92	0,206	0,099	0,376	-0,029
iB_09661	Tc_001099	860	35,03	874	35,60	1025	41,75	849	34,58	301	12,26	1,515	1,538	1,768	1,496
iB_09736	Tc_003166	0	0	0	0	0	0	0	0	0	0	-	-	-	-
iB_09896	Tc_007557	36	0,32	17	0,15	25	0,22	45	0,40	245	2,18	-2,767	-3,849	-3,293	-2,445
iB_09910	Tc_007292	214	7,93	149	5,52	282	10,44	298	11,04	598	22,15	-1,483	-2,005	-1,084	-1,005
iB_09924	Tc_008270	65	2,23	65	2,23	75	2,57	37	1,27	168	5,77	-1,370	-1,370	-1,163	-2,183
iB_09988	Tc_014025	1515	43,32	1535	43,89	1041	29,77	588	16,81	841	24,05	0,849	0,868	0,308	-0,516
iB_09991	Tc_014033	325	22,87	336	23,64	133	9,36	97	6,83	179	12,60	0,860	0,909	-0,429	-0,884
iB_10007	Tc_033075	newly annotated gene													
iB_10104	Tc_013627	295	1,87	205	1,30	130	0,82	651	4,13	10437	66,23	-5,145	-5,670	-6,327	-4,003
iB_10133	Tc_014774	584	9,48	538	8,74	596	9,68	610	9,91	535	8,69	0,126	0,008	0,156	0,189
iB_10156	Tc_015307	3197	52,94	2938	48,65	3400	56,30	4317	71,48	1868	30,93	0,775	0,653	0,864	1,209
iB_10159	Tc_015429	12496	401,73	14782	475,23	19055	612,60	8154	262,14	2891	92,94	2,112	2,354	2,721	1,496
iB_10181	Tc_015547	638	48,39	589	44,67	613	46,50	619	46,95	344	26,09	0,891	0,776	0,833	0,848
iB_10206	Tc_015049	215	2,51	181	2,11	211	2,46	180	2,10	196	2,29	0,133	-0,115	0,106	-0,123
iB_10701	Tc_014985	276	26,09	334	31,57	265	25,05	303	28,64	252	23,82	0,131	0,406	0,073	0,266
iB_10748	Tc_007828	1	0,12	0	0,00	2036	243,30	0	0	0	0	-	-	-	-

bold identified both by the phenoytpic iBeetle screen as well as transcriptomics
