# Supplementary material for: Phenotypic screen and transcriptomics approach complement each other in functional genomics of defensive stink gland physiology
Source: BMC Genomics. 2022 Aug 20;23:608. doi: 10.1186/s12864-022-08822-z (PMC9392906; doi:10.1186/s12864-022-08822-z)
Supplement: Supplementary file 8 — Additional file 8: Supplementary Table S5. Representation of transcriptomics-identified genes in the iBeetle screen. Phenotype descriptions of the transcriptomics analysis are taken from Li et al. [28], iBeetle-phenotypes unchanged form wild type are indicated with 'non detected' (n.d.). Genes that have not been analyzed for a stink gland phenotype as they were only part of the pupal screen of the first phase are listed as not analyzed (n.a.). The 13 genes causing a gland phenotype upon knock-down and were also covered in the iBeetle screen are marked in italics. RNAi-knockdown of genes highlighted in bold resulted in easily detectable and strikingly changed stink glands, and therefore should have been detected in the iBeetle screen. However, only one of the two (underlined) was detected. Changes in annotations between Tcas3.0 and Tcas5.2 are provided. [file 12864_2022_8822_MOESM8_ESM.pdf]

Additional file 8: Table S5 Representation of transcriptomics-identified genes in the iBeetle screen

OGS_# (ass. 3.0)	GT#	categorized phenotype	iBeetle			annotation notes (ass. 5.2)
			iB_#	phase	phenotype	
Tc_000889	*GT01*	*turbid*	*iB_03099*	*2nd*	*n.d.*	
Tc_000917	*GT02*	*melanized*	*iB_00186*	*1st*	*lethal*	
Tc_001937	GT04	wildtype	iB_06143	1st	n.d.	
Tc_002483	GT06	less secretion				
Tc_002669	GT07	wildtype	iB_00423	1st	lethal	
Tc_002938	GT08	wildtype	iB_03472	2nd	n.d.	
Tc_003110	*GT09*	*less secretion*	*iB_03500*	*2nd*	*n.d.*	
Tc_003112	GT10	wildtype	iB_00517	2nd	n.d.	splitted in Tc_032318 and Tc_032319; iB_# refers to Tc_032318
Tc_003284	GT11	wildtype	iB_03534	1st	n.d.	
Tc_003768	GT12	turbid				splitted in Tc_032538 and Tc_032539
Tc_003771	GT13	empty/necrotic				
Tc_003835	GT14	less secretion				
Tc_004085	GT15	wildtype	iB_09573	2nd	n.d.	splitted in Tc_031224 and Tc_031871
Tc_004655	GT17	less secretion				
Tc_005106	GT18	melanized				unmapped
Tc_005384	GT19	wildtype	iB_00841	1st	n.d.	
Tc_005389	***GT20***	***melanized***	***iB_09413***	***2nd***	***conf.***	
Tc_005529	GT21	wildtype	iB_03974	1st	n.d.	
Tc_005635	GT22	melanized				not annotated as gene in iBeetle-Base
Tc_006131	GT23	wildtype				
Tc_006800	GT24	wildtype	iB_06393	1st	lethal	
Tc_007254	***GT25***	***melanized***	***iB_07205***	***2nd***	***n.d.***	
Tc_007317	GT26	wildtype	iB_04313	2nd	n.d.	
Tc_008412	GT27	wildtype	iB_04481	1st	n.d.	
Tc_008413	GT28	melanized				
Tc_008414	*GT29*	*less secretion*	*iB_04482*	*1st*	*lethal*	
Tc_008674	GT77	wildtype				
Tc_008677	GT30	wildtype				
Tc_008780	GT31	colorless				
Tc_008804	GT32	irregular size				splitted in Tc_033804 and Tc_033805
Tc_010236	*GT33*	*less secretion*	*iB_04792*	*2nd*	*n.d.*	*merged with Tc_006847 and Tc_006848 to Tc_031669; iB_# refers to Tc_031669*
Tc_010551	GT35	wildtype				
Tc_010909	*GT36*	*less secretion*	*iB_01760*	*1st*	*lethal*	
Tc_011094	GT37	wildtype				
Tc_011149	GT38	wildtype				
Tc_011211	*GT39*	*colorless*	*iB_01827*	*1st*	*n.d.*	
Tc_011236	GT40	wildtype	iB_04999	1st	n.d.	
Tc_011337	GT41	wildtype	iB_05015	1st	n.d.	
Tc_012298	GT42	wildtype	iB_01963	2nd	n.d.	
Tc_012613	GT43	wildtype				replaced by Tc_034402
Tc_012640	*GT44*	*less secretion*	*iB_05283*	*1st*	*n.d.*	
Tc_012841	GT45	wildtype	iB_05335	1st	n.d.	
Tc_012946	GT46	wildtype				
Tc_013059	GT47	turbid				
Tc_013065	GT48	wildtype				merged with Tc_013066 to Tc_033253
Tc_013108	GT49	wildtype	iB_05385	1st	n.d.	
Tc_013136	GT50	wildtype				

Tc_013434	GT51	wildtype				
Tc_013474	GT52	wildtype	iB_05439	2nd	n.d.	
Tc_013630	GT53	wildtype	iB_08367	2nd	n.d.	
Tc_013755	GT54	wildtype				
Tc_013827	GT55	wildtype	iB_05508	1st	n.d.	
Tc_014185	GT56	less secretion				
Tc_014359	GT57	wildtype				merged with Tc_014360 to Tc_033142
Tc_014388	GT58	melanized				
Tc_014479	GT59	wildtype				
Tc_014748	GT60	wildtype	iB_10470	2nd	n.d.	
Tc_015044	GT61	wildtype				
Tc_015151	*GT62*	*empty/necrotic*	*iB_05763*	*1st*	*n.a.*	
Tc_015346	GT63	colorless				
Tc_015382	GT64	wildtype				merged with Tc_015383 to Tc_033302
Tc_015448	GT65	wildtype	iB_05819	1st	n.a.	merged with Tc_015449 to Tc_033338
Tc_015546	*GT66*	*empty/necrotic*	*iB_05838*	*2nd*	*n.d.*	
Tc_015603	*GT67*	*less secretion*	*iB_05847*	*1st*	*n.a.*	*merged with Tc_015604 to Tc_033405;<br>iB_# refers to Tc_033405*
Tc_015620	GT68	wildtype				
Tc_015886	GT70	less secretion				unmapped; replaced by Tc_031323
Tc_015918	GT71	wildtype	iB_05924	1st	n.a.	merged with Tc_015917 to Tc_033612
Tc_015932	GT72	wildtype	iB_09217	2nd	n.d.	replaced by Tc_033613
Tc_015950	GT73	wildtype				
Tc_016081	GT74	wildtype				Tc_016083<br>to Tc_031339; partly replaced by Tc_031340
Tc_016280	GT75	empty/necrotic				

n.d.	non detected (= wildtype)
n.a.	not analyzed
lethal	no survivors at day of gland inspection
italics	gene knockdowns causing gland phenotypes also covered in iBeetle screen
bold	should have been detectable
<u>bold-underlined</u>	confirmed (conf.)
