# Supplementary material for: Phenotypic screen and transcriptomics approach complement each other in functional genomics of defensive stink gland physiology
Source: BMC Genomics. 2022 Aug 20;23:608. doi: 10.1186/s12864-022-08822-z (PMC9392906; doi:10.1186/s12864-022-08822-z)
Supplement: Supplementary file 9 — Additional file 9: Supplementary Table S6. Non-overlapping fragments (NOFs) for rescreen in 1st phase of iBeetle. In case there is partial overlap with the original iBeetle fragment, this is indicated. Sequence parts of primers used for amplification are underlined. [file 12864_2022_8822_MOESM9_ESM.pdf]

Additional file 9: Table S6 Non-overlapping fragments (NOFs) for re-screen in 1st phase of iBeetle

iB_#	OGS_# (ass. 3.0)	dsRNA sequence (5' --> 3')	notes*
iB_00081	Tc_000379	CCACATTGGTGAGACGGAGCGAAAGTGTTTCATTGAGGAAATCCCCGACGAAACAAACGT<br>CATAGTCAATTACAAAGTGGAGCTTTATGACCCTCGAAGCGGTGGGTTCATGCCGTCGTC<br>CCCCGGGATCGGCATGCATGTGGAGGTCCGCGACCCAGATGACAAGGTCCTCCTGTCTCG<br>AGTGTACAGCTCTGAGGGAAAATCTCGTTCACCTCGCAT	partial overlap with original iB-fragment
iB_00110	Tc_000504	ACCGTGGCCACTGCCCTACAGATTTACCAAAAACCAAGTGGCGACTCCGTCCCAAGCCGT<br>CACACTCGAAACCAAGCTAAAATTACCGAAAAAACGTAAAGATAAACGCCGAGTGAAAAG<br>TAACAGCGAGTCTCAAACGCAAGCGGTTTCATCGCAACAGCTTGCCCAAGATATTGAGGC<br>TTTCATTGGCCAGCCTTTGCAAATGCAAAATAATTCCGGACAGAATTACAACCAAGCGCA<br>AAGTCCCCCAGAAATATCCAGCTCGCAGTCGCAACATAATTTGCTAGTAACCCCTGATAT<br>TCCATCGCCGATGCCCAAATTTTCCTGGGAGACTCCTTGGAGCACCGCTGCTGCGGCCTC<br>GTGCGAGAAATTCGTTTGGTCGCAACCAGCGACTTCCCCGGGGATGAAGCCGATGGTGGC<br>CTCAGCTC	
iB_00185	Tc_000885	ACCAAGCCAGACGGGTCGGTGGAATGCATCGTCGTCCAAAATGCCTACTACCCGAAAAAG<br>TACGTCGATCCGGCAATGATTGGGATCTTGGTCGCTATGTTTCTCATGTTTATTACAATT<br>TGTGTCGTTTTGCGATTGTTCAGCAGGGCACGTTGGAGGGAGAACCGAACCATATTTAAC<br>ACGCCCAACCCGCGCTTGATGAACGTCTCTCTGCTTCGTGAAAACAAGCTTTTACACACG<br>GACCGGCGAGGGTCTCGGAGCAGTGCCAGGGGCCCCAGCAGGCAGCCAAGTATGGCATCT<br>CTAAGAGCTCACTCCCCAAACTCTCAAGGGCGTTTGTTTCTAGGATCTCGTCGCGGATCC<br>CGAGGCAGCAGCAACGCGTCAGCGACTTCGAACCGATCGAACAAGAGTCCGCCCCAAGCG<br>GCCAATTTGAGTTCGGCGACGACGCCCATGCTGGAGAGCGTCACCGTCGAGGTGCAA	partial overlap with original iB-fragment
iB_00414	Tc_002616	GATGGGTTTGCTGGGCTATATCGCCTATGGGTCTGATGTTGCAGACACCATCACCATTAA<br>CTTATCCCCAGAGGATGTCCTAGCACAGGTGGCTAAAATAATGTTAGCCATAGCTATCTA<br>CATCACGCATCCTCTGCAGATGTACGTGGCTATTGATATTATATGGAACGAATATTTAGC<br>GTCAAGATTTGAAAAAAGCCGCTACCAGCTGTTTTTCGAATATGCTGTGCGAACTG	
iB_00754	Tc_004698	GAGACACCAAAGACAAGCCCAGCCCCCGCGACAGGGCTCCACGCCGCCTGCCTACAACGG<br>AAACAACTTCCAGGGAGATCCCCCGACTTTGAATCCCCCCAAAAACCTTTCGCCCCCCTC<br>CAATGGAGACAAACTAGTCCAACCAACACCAAATGTGATCCCGAGCTGCGCGAGGGACAT<br>AACCATGCCCGTTTCGGGGCAAAATTCAGCAGACATGGACGTGAGTGAGGAAGAATTGAA<br>GGACTTCCTCTCGCAGAAGGATCTAGCAACGACGCTC	
iB_01044	Tc_006408	AGCAACAACACCAAGTTCGGAATAGGGGCTAAATACGACTTGGATCAAGACGCCGCGATT<br>CGCGCCAAGGTCAACAATTCCAGTCAGATCGGTTTGGGGTATCAGCAGCGTTTGCGTGAA<br>GGTGTGACTCTAACTTTGTCGGCCCTCATTGACGGCAAGAACTTCAATAACG	
iB_01236	Tc_007650	TGCGGCATTTTGACCCTAATTGCGGCAACGTATCGCAAAGACGTTCGAGTCACTAGTATT<br>GAAGAGAACAAAATCTTGTACGATTTAACCGATACTTTGGTCAAAAAACGTAATTTCAAT<br>ATTCGCCTAGTTAACGGTAACTCTAACACGATTACAACGCGCCCAGCGCCCTGTAATTTG<br>ATAGTAACGGAGATTTTTGACGCG	
iB_01372	Tc_008608	GCTTGGAGACGGCGTTACTTTTGACACAACAACGACCAAACAACTGGAAGAAGTGATCGA<br>AAAGAATGATTTCATCGTTTATATCAATCACATCAAACCCAAACGACGAATTGTACATAA<br>TGCAGAGACGCTTTTTGCGGTTGAATTTCCAGGAACCGATCAGAAATTTGAGCTTAAACT<br>TGATAGAAGCACGAAGCGAGTCATTGTTGAAACTGTGGAGGATGACCGTCGCCGAATCCA<br>GCATTTCAAAGTTGACTCTCTTCATGAGAATAGCATCATCAAATCGCTGATTCTGGCCGT<br>AAATCAGACACAACCAGGAGCACATG	
iB_01440	Tc_008936	CAAATCGGCGACTTCCTCCAAGAAATCGTCCAAAAAGTCTCCAAAAGTTCAAAAGAACGA<br>TAACAATAACAACAGTAGTAATAACAGCAATGCAATCGTGAAAGAGAGATTGTACGCCAG<br>ACGCTCCGCTTTGCCCGAATCGACGAGTTCGAAGTTGAAAG	partial overlap with original iB-fragment
iB_01644	Tc_010033	GTGTCCTAGTCGGCGAAGGTGTCCTAGTTAAAATGTGCCGAAAGAAACCGAAAACCCGAC<br>AGTTTTTTCTATTCAACGACATCTTAGTCTACGGCAACATCATAATAAACAAGAAGAAAT<br>ACAACAAGCAACATATCATTCCCTTAGAAGAAGTCAAATTGGAAAATCTCGAAGATGACA<br>ATCGTAAGTGCCCAAACAAGTTT	
iB_01798	Tc_011075	TGATGATCTGCACCAGGTTGGCCGGAAGGGCCGTCATCAGAGTGGTCGGCCGGTGCAACG<br>AGGCCCAAGGAAATAACAATCTCGATCTCTCAAATTGCCAATTGATGCAAGTCCCCGACG<br>CCGTCTACCACCTCATGCGTCACACCGAACTGAAAACCTGTGATTTAAGTGATAATGTGA<br>TTACGAAAATACCTCCGAAATTCGCCGTTAAGTTCAGCTCGATAACGGACTTGAACTTGT<br>CGCACA	partial overlap with original iB-fragment
iB_01814	Tc_011159	AAAAATCGCATCTGCCAAACAAATGACAGTCAGAGACGCCCTAAATTCAGCCCTAGACGA<br>GGAAATGACCCGCGATGAGCGGGTTTTCATCATCGGCGAAGAGGTGGCGCAGTACGACGG<br>CGCCTACAAGGTCACAAGGGGGCTGTGGAAGAAATACGGCGATAAGCGAGTCATCGACAC<br>GCCAATCACAGAAATGGGGTTCACAGGTTGTTGCTTAAAAGTGATTCGTTTAAAGGACTA<br>ATTTTTGGCCCCCCAGGAATCGCCGTGGGGGCTGCCATGGCCGGCCTGCGCCCCGTCTGC<br>GAGTACATGACGTTCAACTTCGCCATGCAAGCCATCGACCAGATCATCAACTCGGCGGGA<br>AAGACCTTCTACATGTCCGCGGGCCGCGTCAACGTCCCCATTGTCTT	

iB_01910	Tc_011969	AGACACGGTGGTTGAGCCCTACAATACTGTAATGTCGATTCACCATTTGGTCGAAAATAC<br>CGACGAGACGTACATTATCGACAATGAGGCGTTGCATGACATTTGTTTCAGGACGCTGAA<br>ACTTTCAGCGCCGACTTTAGCCGATTTGAATCATTTAATCTCAGCTGCAATGTCTGGGAT<br>AACGGCTTGTATTCGTTTTCCGGGGCAATTAAATGCCGATTTAAGAAAGATCCA	
iB_02292	Tc_014494	CCCATCCGATCATTGAACCGTCGGGTTTTTTGGTGCCGCAAATTTTAAACCAGTATGGAG<br>AGGACTTCATGTTCCTCACTTGCATCCAGTACATAAACTCAGTAAAAAGCGGCCCTTTTG<br>CCGAGCACTCGAACCAGCTTTGGAGCATAAGCGGAGTGTCCTCCTGGACGAAAATCAACG<br>GGGGCCTAATTAAAATGTACAAAGCGGAAGTTTTGAGCAAGTT	
iB_02297	Tc_014520	CGACGCACATGAGAAGGTTATACCAAGGGGGACCTCGACTCTTTTGCAGTTTTTCGAGAA<br>ATAACCTCCCGTCAGAAGTCACCACGAAATACGCCCAAAGCACCGATTTTCTCCTCAACA<br>ATCTTAAAAATGCCAACAGTACCAAGAACGTCTTGGACATGGTCTCGCAACACAATGACA<br>TCATGAACAACAAACACGTTATCCAAGCTTTACGTAGCTTATTCACTTTACAAAAAAACG<br>GAAATTCAGAAATGTCCACAAATGAAATTCTAGCCCACCAAGATTTTCGCAAATTG	
iB_02301	Tc_014544	TTTTCATCGCGTGCAAATACGAGGAGATGTATTTCCCCGAACTTTCAGACTTTGAATTTA<br>TCTGTGATAATTCGTTCACAAAGAATCAGATTCTGCGGATGGAAATGAGCATTCTATCGT<br>CGCTGAAATTCGAGTTGGGCAAACCTTTGTCAATTCACTTCTTGCG	partial overlap with original iB-fragment
iB_02367	Tc_014967	TAGTTTATCGGGGATCAGGGATTTCACTAACAGTGGTACCATCTACTTCCCCCATTTGCG<br>GATTTCGGTTGAGAAACCTCGGGAGCAGTCGACGTTGGGCCAAAATCGGCGAAATATTAG<br>TGGTAACTCGGAAGTGTTCACAAGTGACGAACTTGGAGTGGCCCGAGAGGAGGTTTATCC<br>CGCTGTAGTTGAAAAACAAGTTGATTTAATTAGGGAGAAAAAAAATAATCGGCCAAAATT<br>CAGACCGGATTATTGGGACCACAACTATGCC	
iB_02401	Tc_015095	TGTGATCCAAGCAGGCATTAATATGTCGTTCAGCGCCATCTTACTACCGCAGCTGAACGA<br>AAAGTCGAGTGATATACATATTTCCAAATCAGAGGCGTCTTGGATCGGTGGGTTTAGCCC<br>GGTTATAGCCGATTTAACCGTTGTTTTTAGCAAGTATAGTCGCAATAGCCCTTCCAGCGG<br>GTTCGTTGATCATCGGCCCGCTTATGGACCGCTTCGGGCGCAAAACTTTGTGTATTTGTA<br>CGACTATACCCTTCGCCATCTCGTGGATAATC	
iB_02416	Tc_015165	AAACACGCGCGCTACTTAGAGAACGAAATCGACTTAAAACTCGTCGCGTTTAGTAAACTG<br>GGGGCCGGTATCAAGTCGCCCCCAGCTCATTCGAGCTCCGATGCTGTGCCCCTTCTCTCA<br>GGCGAGGACACTTTCGAGGCCATGTCTCTCGAAATTGAGGAATTGTTGAATAAAGTAAGC<br>GGGCCCCCAAAAAATCACCCCAAATAACAATTCCTT	partial overlap with original iB-fragment
iB_02428	Tc_015203	GTTATAGGCAAATGGCCCAACACCTACACGTTCACCAAAGCTTTGGCCGAAGCTTTGATC<br>AGAAACACCGCCACGAGTTTGCCTGTCGGGATTTTTCGACCAGCGATCGGTAGTAGAAAA<br>AATTTGTATGTCATCCGTGAGATAACTGTCTATTTTAGTTATTTCAACGTACAAAGAACC<br>AATGGAGAGTTGGATTGATAATTTGTATGGACCTACGGGGGCTGTAGCTGGGGCTGCTTC<br>TGGACTTCTCCGTGTTTTTCCATGTAATGAGGATGTGGTGGCTGATATTGTACCAGTTGA<br>CACGTGTGTGGCCGGAATTATCGCAGCGGCGTGGGATGTGACAAATAA	
iB_02471	Tc_015379	AATGGACCGTTGTTCGATTTATTGAAGAAACAACAAGGTGATGAAATTTTAAAGAAAGTT<br>GAAGCAATTTCTGCAGACATGGAAGCACCTGATTTGGCCTTGGCCGCCTCTGATCGGAAG<br>AAATTAGCCGAGGAAGTGGAAATGATTTATCATTGCGCTGCGACAATCAGATTTGATGAA<br>TCTCTTCGTAAAGCCGTGTTTCTCAATACTAGGGGCACTAAACTAATGCTTGATTTGGCT<br>AAAGAATGCAAAAAATTGATCGTTTTCGCCCATTTGAGCAC	
iB_02516	Tc_015811	CCAAAGCCGACCAAGTGTGGGACCCCGCCCTCCCCTGGCAGCCCATCCCAATTCACACGA<br>CCCCCGAACTGGAGGACAACCTCCTCTCAATGAAAAAAAACTGCCCCAAATACAACTCGC<br>TTCTAACCCAACTTTTCAAAACCGAGTTTTTCGCCAACATTAGTCGACAAAATCGGGACT<br>TGTACGCGTATTTGAGCAAAAATTCAGGGGCCAATATAACGTCCCTCGA	
iB_02517	Tc_015818	CCCCACAGGCGACTACACCTCCGACCAACCAACAATAATCACAGTGTCAGAAGATGACAC<br>GATTAAAATCACCGTCTTGGGCATGACGTGCCAAAGCTGTGTCAAAAACATCGAAGAGAC<br>CCTGAGTCGTAAACCCGGCATTTACAACATCAAAGTCAGCCTTCAGGAAAAAGCCGCTCT<br>AGTCCATTATGACACACGCCAACTGACA	
iB_02542	Tc_015993	CCAAAACTGCCCGTCTATTCAAACCCCGTATTATTATCGCCGGTATCAGTTGCTACTCCC<br>GTCCTCTGGACTACAAACGTTTCCGCGAGATTTGCAACGAAGTCGGTGCCTACCTCATGG<br>CCGACATGGCCCACATTTCCGGTTTAGTTGCTGCCGGCGTCACCCCAAGTCCCTTCGAAT<br>ACGCCGACGTCGTCAGCACCACCACGCACAAGAGTTTGCGAGGACCTCGAGCCGGTGTCA<br>TCTTCTTCCGAAAAGGCGTGCGGTCGCATAACGCTAAAGGCGAACCCATTATGTATGATC<br>TTGAATCGAAGATCAACCAAGCTGTCTTTCCC	
iB_02563	Tc_016254	CCGGAACGACTTGATCGTAAACAGCAAAAATTTGAATATTGAAGCGGTGCATTTGATGCG<br>AGATTGGAAAAGTGTTGAGATCGATAATGTGGAGGAAAATGTCGTTGATGAGGTTTTGAT<br>TGTTGAAAGTGAAGAGATTCTCTATCCGGGGATTT	

iB_02584	Tc_030051	GGAGTCCAGAAGGCGAATTGGTATGGGTCTGTTACCCAGGCTAGCACGGTTAGATTGGGTGGCGATGTCTACGTACCATTGGGCAAGTTGTTGCCAACGGTACATCCCGACGATTTGGTGATTGATGGGTGGGATATCAGTTCGGCCAATTTGG	
iB_02625	Tc_011255	ATCGATGAGCTGTATGCTGAAATTTTGTACGAAATCTTACATAATGTTGGCTGTGATGTAAGTGTTGAGATAGGACAGACTGCCTTAATCTCGTACGCACAAGATGCATTTAAAATTCCTAACAATAAACATAATCAACTATTGTCTGAAGCTGAAAAGAAGGAGGCTCCTGAATTTTTGATCAATGTCGAAGTTATCGAAGCCAAAGATTTGAAACCAAAGGATTCCAACGGGTTGAG	
iB_02627	Tc_011288	CAGACCTTGAACTGGAAGGTCTCTTCAAGAGACATTTCACAACTGTAGAGTTCTTCCAGGGTACCATCATGAACCCTATTGACCTTCAAAGGGTAAAGGTACACGAAGCTGATGCGTGTCTAGTCCTCGCCAACAAATACTGCCAAGATCCAGATGCTGAAGACGCAGCCAACATCATGCGCGTCATTTCGATTAAGAACTACAGTGATGATATTAGAGTTATCATCCAGTTGATGCAGTACCACAACAAGGCTTACCTCCTGAACATTCCATCATGGGACTGGAAGCAAGGCGACGACGTAATTTGTTTGGCTGAACTGAAGCTAGGTTTTATAGCTCAATCCTGTTTAGCGCCCGGTTTTTCGACAATGATGGCGAACTTGTTCGCTATGCGTTCGTTCAAAACGTCTCCAGACATGCAGGTGTGGACCAATGACTATTTGCGAGGCACGGGAATGGAGATGTATACCGAGACGCTCAGTCCGTCGTTTATAGGCATGC	partial overlap with original iB-fragment
iB_02633	Tc_011371	GCCGTTTACGACACCTGGGGCCGCAAAGTCCCCGGAAGAATCATCTTCTTCTCGTCGGAGGGCTCCACCAGCGCCCACGTGCCGCTCGTGGCGCTGCCCCACGTCGACGACGCCTACCCGCCCCAGAAGAAGTCCTTCCACATGCTCAAGTACCTGCACGACCACTTCGTCGACGAGTTCGAGTGGTTTGTGCGCGCCGACGACGACGTCTTCGTCAAAACCGAGCAG	
iB_02673	Tc_000240	AGGTGTGACAACAATGCTCAACTTTTTCACAACCTCAAACGGATTCCGTTCAACTTTGCCGGTGGTTTCGAATCTGACTGCCATGAATGTGTGGGACGGTGTGTGCATGTGTTTCATTTACGCGTCCTTATTAGAATTCGTATGTGTGAATTACGTAGGAAGAAAACGACCGCTTCATAACGTCGTCT	
iB_02692	Tc_003063	ACTGGTGTGCTACAGGCCAACCAAACGTGGACAAAGCCACCTCAATTTCCCAACTAATCAACAAGCACCAGGAACAAAAGGAAGCCTTCCTCAAAGCTTGCACTTTGGCTCGAAGAACAGCCGAGACTTTTCTAAAATACACAACTCGAAGTCTCCAATTCTACAACTACTCCAACACCACGAGCAATCACGAGACCAGAGTTAAGGGCATTTTGGAGAAGCT	
iB_02716	Tc_002723	GCCTCGTCTCCTTCACCCCCGAGCAAGCCAAACCCCACTTAATCGACATCAAATTCAACGGAGAGACGGTGCGAGGATGCCCCTTCGTGTGCTCAGTCGCTGACACAAGCCGAGTGACTTTGAGTTTGAGTCACTTGGAGTTAATCCCCGTCAACCAAC	
iB_02931	Tc_011812	CCGTAACCAGGACCAGATGAAGGAGATCATCCACGAGATTGCCGTTTTAATGCAGTGCTCGTCGACGAATCGCGTGATTCGTCTGCACGAGGTCTACGAATCGGCCACGGAGATGGTGTTGGTGCTGGAATTGGCCGCCGGCGGCGAGCTCCAGCACATCCTCGACGGCGGCCAGTGTCTGGGCGAGGCGGAGGCGCGCAAAGCCATGAAGCAGATCCTGGACGGAGTGTCGTTTCTCCACGAAAGGAACATCGT	
iB_03401	Tc_002550	CCAACTCCACCACCACCTCGCCCCACTCGCTCAGCCCCAAGGCGTTCCAGTTCGACACGGTGGTGCAAGGGGGCAGCGCGCCGCCCTCCAGTAAAGTGTCGCCGCTTATCAGGGACTTTGTGCAAGCCATCGACGATCGCGAGTGGCAAAACTCTTTGTATACTTTACTCCAGAACCAAACGTATAATCAGTGTGAAGTGGACTTATTTGAACTTATGTGTAAAGTTTTGGACCAAAATCTGTTCTCTCAAGTCGATTGGGC	
iB_03552	Tc_003409	TGAACCAGCTGTGCAAGGTCTGCGGAGAGCCAGCGGCCGGATTCCACTTTGGAGCTTTTACCTGCGAGGGATGCAAGTCCTTCTTCGGTCGAACGTACAACAATATCAGCTCAATATCGGAATGCAAAAACAACGGCGAATGTGTGATCAACAAAAAAATCGGACAGCTTGCAAAG	partial overlap with original iB-fragment
iB_03637	Tc_003857	CATAAGGCCCCTAATGAGCAACGTGTTAATGGTAGGTGGGGGAACACATTTTGAGGGAGATACTCCCCTACCTGAAGATATACAAAAATTTTTGGACGGGGCTGAAAACGGAGCGATTTATTTCAGTCTTGGGACAAATGTTAAAAGCAAGGATTTGGACCAAGACACGAAAACAACATTTTTGCAAGTATTTTCCGAACTGCCGTATAAAGTGTT	
iB_03693	Tc_004126	TTCGAAAGCGAATTTGAAACGAGACAAGAACAGCTTGAGTAAAAGCAAAACTATCGGAAATTCAATTTTAGACCACAGTGATTTTATTAGTCACTTGGACGACGACGAAATTAGTAATAATTCATACGGGAGTCACACTTATCGCTTTAAGGACGAGAGTCACAAAGGCAGTGCCGACGTTCTTGAAGATAACGAAGAGCAGGAAGAGAAGCGC	
iB_03695	Tc_004129	ATGGACCACATAGGCGACATGATCGAGCAGCAGGCACTGGGTGATCCGGTCGATTCTCCTGATGGCAGCGCGCATGCGGCTGGTGGCGGCGGCATGGAGACGCAGACCGTCCGCATGAGGTTGGTGTCGCGTGCTGTGGTGGAGAGGCGTCTTGTAAAACAGGAAG	
iB_03780	Tc_004533	TGCCTTTGGACGAAAGTAAAGACACACTAGGGGGTAAAAAAATCCGATTCAAGACCCCTGCGCGTGTTTTGTTCACTTCCGAGAGTAAAATCGACGACTTATTGCGAAATGACAAGGAGGCGCAAGAAGGGGAGGCAAAGAAAGGTAAACATTATAGTTTCAGGGGGCTCTCCAGTGTTGTCATTGCTTTTAGAGAGAAGTCCATCCCTGGCATCGTTCTTACAAGTGGAGTTGACCCGTGACT	partial overlap with original iB-fragment

Additional file 9: Table S6 **Non-overlapping fragments (NOFs) for re-screen in 1st phase of iBeetle**

iB_03913	Tc_005167	AGCTTTCCACCGAAAATGGAAAGGTCCCAGATAGTGAATTCGATGGGTTCATCCGACCAGTTTCAAGAACAGACTTACATCCAGGCTCCCAGTTTTGTCCAAGCTCAAAACTACAGTACTCCAACCCCCAGTCCTATCTATTCCCGTGCCTCC	
iB_04066	Tc_006098	CTCCAGAACCCGAAACTCCCAAACCAACTCCCTCAGAAACTCCAGAGCCCGAAACTCCAAAACCAACTCCACCCGAAGACAGAGATCCAGAATGTCCTTGGCCAGATCCTTTGGATCATACTGTTCATCTACCACACGAAACGGATTGTACGAAGTTT	
iB_04137	Tc_006423	GTGCGTTTGTCAACGTCTCATGTCGTGCTGGTCTGGAGATGAAGACATAAACGCCCTTTATGCCACAGTTGACAGGAGTAAAAAATGTCAACGCAATCAACCCACAGCTGCCAACTACGCCAACATAGCTCCCATGTCGGCCCCCACTGTTCCTACTTCTTCCAATTACGAAAACATGGAATTTGCTCAAACTCTCAAACTGTACGAAAACAGCAAAGAAATCGTGGAAAA	
iB_04205	Tc_006735	GTTAGAGGCGAACACGGAGACAATTTCCCCTTTGACGGCAAAGGAGTGATTTTGGCGCACGCTTTTTTCCCAAACGGTGGCCACAGCATCGACGTCCACTTCGACGCAGATGAGGCCTGGACAACTGTGCCCAACAGCGATGAAGGGACTAATTTATTCAATGTGGCGGC	
iB_04797	Tc_010251	CCTTCGAAAATATCCCCCAGGTCAAACTTTGAATCGCCGCTGTGTTAAAGACTACACCCTTCCAGGAACTAGCACAATCATTGAAAAAGGGACTCCCATCCTTATTTCTGCAATAGGAGTTCACAGGGATCCTGAGTATTATCCAGATCCTGAGAAATTTGATCCTGAGAGGTTTAGTGAAGAAAATAAAAAATTGAGACATCCGTTCGTTTATTTGCCATTTGGGGATGGACCAAGAAATTGTATTGGAATGCGATTTGGAACTATGCAGTCTAAGTTAGGGATTGCATCAGTTGTGAAAAATTTTAAAGTGTCTGTTAGTCCCATAACAAAGCGGG	
iB_04839	Tc_010449	GTGTCCACTGTTACTCAACCCAGGACGGCAAAAAATTAAAAAGGAAAGTCAAAGCCGAAGGATACGTCGAATGCTCTGCCAAAACCAGGGAAGGACTGAAGGAAGTCTTCGAAGAGGCGATACGAGCGTACAAGAAGACCAAAATCAAAGCGAGACAAGTCAACTGTGC	partial overlap with original iB-fragment
iB_04850	Tc_010484	ATAAGGGCACGCAAATCAAGGGACAGGATTCTTGATTTGGAGGACGAAATGGCCGCAATGACCGAAAAACAAGCAGCAAGGGAGCGCAGAGCGGCTAGGTTTAAGGCATTGGTTGCTGAAAGCACGGAACAAACTGAAGCGGCACAATCGGCCCTTCAAGCGATTAGCTTTAAGTCCAGGAGGGAACAAAGGACCTTGGAGGAGTA	
iB_05264	Tc_012539	GTCCAATTGTCATGGGTTGCACCATTCGATGGCAACTCCCCGATCACTCGTTACATGATCGAGTACAAGCAAAGCAAAGTCAGTTGGGAGGGAAACACCGAACGGGTACTAGTCCCAGGTGACCAAACTGAAGCTGGGGTTTTCACTCTGCGACCTGCCACCACGTATCA	
iB_05278	Tc_012610	CACTGCAGGGACTGGCACGGCTGCATCATGGCCCAGGCTATCATCGGACAGCACAACATCCAGCCGTACAAGTTCTCCGAATGCAGTCGCTCGGATTACATCGACAGGCTGAGGACCGGGAACGGGATTTGCCTGCTGAACAAGCCCAACGAACTAGAGG	
iB_05284	Tc_012642	GGTTCTCCCGATTTTCGCTATAGGGATTATTACAGTTTACCGGAGAGTTGCTGTCCAGTTAATACGACTCACTGTTACCACACAAATGCCCACAAAAAACTCTGCAGTGTTAAACGTTTAACCATTTGCGACGCAACCAAAGAGGCCTATTTGAGGAGTTACGTTATGTTACTGTTCATTCCTCTTATTGTAGCATCTGTAAGATTAAAAAAACTGGAGAACAAGTCCTACGCTTTTTTACAGGTTGTGCAAATAGCTCTGA	
iB_05329	Tc_012828	CCAATACCACCACCCCCTGTACTACCCCCAGCATATCCCCGTGCTCGACCACAACGGCGTCCCTGTCGAACCCGCCGCCAACCAACTCGCCCGCGCCGCCCATTACGCCGCCCATGCCGAAGCCAATGCTCGCACTGGTCATTATCCAATTTACGCCGCC	
iB_05331	Tc_012834	TACGTTGCACAGTTTCGGAGCAAAGTGTTACAGTTCGGAGTTTTTGGCTTATGACGTTACGTGTGATACTTGGCAGGTGTTACAAGTGCCAAAGGATATTCACGCTGATTTGGCTCGTTTTGGTCATTCTGCCGTTACTTTTGAGGGTTCGTTGTACATTTATGGAGGGTTTGACGGACAAATGTTGT	
iB_05342	Tc_012857	GTACTTAACGGAATTGGCCCCAGGTTTATTAAGGGGGTCCATGGGGGTGTTGTGCCCCCTTGGCGTGACTTGTGGTGTCCTATTGGGGCAAGTTTTGTCCTTAGAAGGGATTTTAGGGAATGAGGATTATTGGCCCCATTTGTTGGCCTTCTATTTGCTACCTCTTGCTTCATGTAGTGTGATATTAGTATTTTTGCCTGAAAGTCCAAAGTATCTTTTTATAATCAAAAAGCAACCACATCTAGCG	
iB_05442	Tc_013513	TTGACAACAAACGCTATCGGTACGCTTACCACCGGTCCTCGTGGCTGGTGGCGGGCAAGGCCGATCCTCCGGCTCCCTGCAGGATATACGCGCATCCGGACTCGCCCTTCTCCGGGGAGCAGCTACGAAAGCAAGTCGTCTCCTTCGAGAAGGTCAAACTCACCAACAACGAAA	complete overlap with original iB-fragment
iB_05518	Tc_013892	GCCGTTTCAGCCTGTTTCTACATTCTTGCAATTCAAAAATCCGAATCAAAATTATTTAACCCATCTCTAGTTGTCAGTTTTATCAAGCCAACAATTGTAGATTTGATTGATAAAAGTGTTGAAGAATCACGATGTGCCTCAGACCTAAAACAATTCATCACCGACTTGTTCAAACTCAAATGGTGGGCGATTCGAACTGAATACATCATCCCTTGCTACAAAGCAGACCCCGAAATCAACAAATGCCTCCAACG	
iB_05584	Tc_014205	AGGACGAACTGACGCTCTTCCCCTCGCAATACAGCATAGACGTTCACGGCTATGTGCTCGTCTACAGCATCACCGATATCCGGTCGTTTGAAGTCGTTGGGACAATCTTTCACAAACTCCAGGACCTCAACGGGAAAATTCAGTGAGTTTGGCCCCTTTTGTGAGGCTGAGGCTAGAGCTTTTG	complete overlap with original iB-fragment

iB_05712	Tc_014870	CGCTCCGTGACAACCAATCGGCGCACGTCACGTGACGCCGCGTGTACCATGATAATTGGTCGAGACACCCACCAATTGGGCCCCAGCGCAGTCCGAGCCTCTAACATAACCTCAACATCGGCTGTCATCAGTTGGCTACCAGCCAACTCGAACCA	
iB_05719	Tc_014887	GCCAGTGCTGGATGTGGTGCCGCCATCGCCGAAGCCCTCGTCCGCGAAGGCCTCCAGGTGGTGGGTCTCGCCCGTCGCAAAGCCCGCGTCCAAACCCTCGCCGAAAAACTCGCACCACATCCCGGCAAACTCTACGCCGTAAAGTGCGACATGACGGTAGAATCTGACATTTTGGAGGCCTTCAAGTGGATCAAAACCACGCTAGGGC	partial overlap with original iB-fragment

* partial or complete overlap with original iB-fragment: e-rnai web service resulted in no alternative sequence to use and/or iB-fragment already spanned almost 100% of gene length
