# Supplementary material for: Phenotypic screen and transcriptomics approach complement each other in functional genomics of defensive stink gland physiology
Source: BMC Genomics. 2022 Aug 20;23:608. doi: 10.1186/s12864-022-08822-z (PMC9392906; doi:10.1186/s12864-022-08822-z)
Supplement: Supplementary file 10 — Additional file 10: Supplementary Table S7. Non-overlapping fragments (NOFs) for rescreen in 2nd phase of iBeetle. The NOFs were ordered ready to use from Eupheria Biotech GmbH (Dresden, Germany), which also determined the best sequence. [file 12864_2022_8822_MOESM10_ESM.pdf]

Additional file 10: Table S7 Non-overlapping fragments (NOFs) for re-screen in 2nd phase of iBeetle

iB_#	OGS_# (ass. 3.0)	dsRNA sequence (5' --> 3')
**iB_00105**	TC000476	TCGCTGGAAAAGTATCCACACTCAGCAAATTTATGGATAAACATGGAGCTCTTGGCGAATTCTTGTTTGATAAGAAACTCCTCAGTGGAGAATTG
**iB_01975**	TC012387	ACTGCGAGATGGAGGTGTTCATGTCCGCAGTCAGGTGGCTCAAGTACGACTGGGCCAACAGGGATAAGTACAAGTATGAAGTGCTCAAGTGCGTCAGATTCGGAAACATAGCCGCTTGGCAACTTGTTGATATCAAGCGCAATCCCGAAAATCCCGAATTTATGGAGTTAGCCAAAGACCCAGCCATTTGCAAGCTCATCGATGACGGGCTGGCGTTTGTGATCATCAAGCACTGGTACGACCAC
**iB_02774**	TC008303	GCAACCATGTGAGGAAGGATCCGATGCCGATTAGTGACGATAGTGTTATTTATATAGACAGTGATGGGTTTAATCCGATGAAGAAAAGTGATGATAATTACTGCCAAGTTATAGACTTATCTGATAAAGCCGAGTTCAAGAAAGAAGCGAAGAGTGTTCCAAAGAAGGAGACTAGTATCAGTCCGTTGATTCCCGAGCACGTGTCGAAACATCACGAACTGAGTATTACCCCGACTTACCCCAAAATCGACCC
**iB_03294**	TC002074	GGAGAAGGATCGGGAGAGTCGCTGTGGTCACATGGAGACTTTTCTTCAAACCTTGATGGGGTTCGAGGGTGACGATGACGATAATCTACCAATGATCGCAAAGACTTTGGCCAACATAATCCAAGACGATCACAAGTGCGGTGAGCTCAAGCCGGAACAAATGACTGACGATGAGCTGCTTGAAAGCATCAAGTTGCCACTGTTTGTCCTCTTCCAAACGCT
**iB_04702**	TC031200	ACGTATCGAAGCGGAAAAGAAGCTCCTACTTCCCGAGAACGAACACGGCGCATTTTTGATCCGCGATTCGGAAAGCCGGCACAACGATTACTCGTTATCAGTCCGTGATGGAGATACCGTCAAACATTACAGAATCAGGCAACTGGATGAAGGTGGTTTCTTCATCGCGCGGCGAACGACGTTTAGAACGCTTCAAGAACTAGTGGAACATTACAGTAAAGACCCAGACGGTCTGTGTGTCAATCTGTGCAAACCATGC
**iB_04717**	TC009877	AACTGCCCAACCACTTCTTGTCCGAAATTTACAAATGGGCCTTGGAATCTGTGGCTAGAGTCTCTTTGGACACTAGACTTGGCTGTCTAGAACCGAACCTGTCCCAAAATTCCGAATCACAGCGAATTATCAATTCCATAAACACCTTCTTCTGGAACGTGGCAGAAGTAGAACTGAAAATGCCCGTTTGGAGGGTTTACAAAAACAGAAGTTTCAAAAAATACATCGGTGCTTTGGAAGAC
**iB_06359**	TC031191	TAGTCTGGAGCGAAGCTACAAGAGCCCCTTGTTACCAGAGCGGGCCCTCACTGGCCGTTATGACCCAGTTAGCGACATCA
**iB_06806**	TC000393	ACGCGCAGTATTCGTAGCTTAAGGATCCGAAAGCCGAAAGAACTTAAACTGCTTTTGAGAGAAATCTGGGAGCAGACCAAGGCCTTGTGCAAGCCGCCGCATCTCAGATATACCATAATTACGTGTCTGATCCAATTCGGCCTAACTACAAGTTACTACACTTTGATGATTTGGTTCCCGGAGCTTTTCTACCGGTTTGAAGAGTTCGAAAATCTTCATCCGAACGAAAAAGCCACAGTGTGTG
**iB_07361**	TC009459	TGTAAACCGCGATGAAACAAATTTATTGTTTAATAACCGTCGTCGTTCTAATCCCGACACTCACTGCTCTTGAAAATGAAGGGCAAAATCCTGATACAGCAAATTGTGTTGCGCTAGGTGGACAAAGAATCAAAGATTCTGAAATAGCAAAAATGGCTCATTGCATTCTCACCAA
iB_07902	TC001275	GGACAGAAAGGTGAGGACGAAGTGATGAATCAGATTCTAATAGAATGCATGAAACATTACAACCTGATTAAAAAATACACCAGTTTAGTAGCAGACTGTTTCAAAGAAATTATAACTTTGCAGTTCGTTCCAACGATTGTAATGATTTGCATTGCAATGTATAAAATATCCACGCTCGAGCCGTCGAACACGCAGTTTTGGTTTTTTGCGTTTACGGAACTCGGTGCTATAACCCAAATTTTCATTTACTGTTTCGTTGGCAATCTGGTCACTA
**iB_08398**	TC033206	CCCCAAAACGTTGATATTGCACGTGATAAGTTTCTAAGCGAGTTGCGGAGCGCTGAAGCGAAAAAAAACGAAAGTTCGCTCCACAACTCGTCTCCCTTGTTCTCGCACGTTTCCAAACCCCAAACCACCAAAGAGTTCACCAGGTCCGAGCCGGACTTGAACCTACCGGATAATCTAACCTTTGCTAACGCACTAACAGAAGACAAAGCTAAAAATGCTACCCCGGTTTGTTGTCAGT
**iB_08760**	TC033755	GACGGTTCTCGGTTGAGAGAGACCCTCAAGCTCGATTTGAAGAATTCCGGGCTTTGTTAGAGCGCTTGCACAGACTGCAGGACATAGCATTTTTGGTTTCGTATATTGACCCGAGCGATCAGGATTTGCTGCCAATCAACAATGATGATAACCTTCGAAGGGCCTTAGCAAATGCAAAACCCCTCCTGAGGGTCATAATCCAAAGAAAAGGTGATAGTCTTGAACTGAATGGATACGGGA
**iB_09043**	TC016314	GTCTGGGAATGGACTCAGGATAATTGGTTGAATGACCCTGACGCCAAAGTGAAGAAAGGGGGCTCCTACCTCTGTCATGAGTCTTACTGTTGGAGGTATCGCTGCGCTGCAAGATCTTTCAATACCAAAGACAGTTCGGCCGGAAATTTAGGCTTTCGATGTGCCGGTGATGTCAAATAATTTGGCAGTGAATAACAAACAAACCCAAGTACTTAAATCATCCTGCCATCAAGTCTTCC
**iB_09050**	TC033022	ACGGGGTTTAGTGTGGAGTGTCAAGTGGACAGGTCTCAAGTGAAAAATCGTCAAATTGCTTTGGCTAAGTTGAGGGCTTTGATTTATCAACGTGATCTTGAAGAGCAGATAGCGAGGAACGAGAATATGAGGAAGAACCAAGTTAGGTCGAATTTTAGAAACGAGAAAATTAGGACTTATAATTTTCCGCAAGACAGGATCACTGATCATAGGTTACAGGGGTGCAATGT
**iB_09103**	TC014482	AATGGGGGCGGGTATTATCACTATTCCTATTCCGTGGTCAGGGGCTGTAACCGGATTATTCCGGTCGATATTTACGTCCCGGGGTGTCCCCCAACCGCTGAGGCGCTCATGTATGGGATTTTGCAGCTTCA
**iB_09413**	TC005389	TGCTTGATTTTTCCCTCAGCTGATTATGTCCGAAATTTGGTGATTAAGCAATCGATGAGGCAAAAAATTCCGGTTGTTATCGACTGTTCACACATTTATGGTGCTGATTATACGGCAGCTACAGTCATTGAGAGTTTAACCAAAGATTTCAAAACGCGACAACAACCGCTGTTTTTTTACAATCTCAAGTCAAGTGTGAGCTCGGTTTTTGTCGGCCTAAACCTGGACTATTTTCTAGTCTATTACAACGAAGATGAGTTGGATGATATGCTCAAGAAGTGGACCGAAA
**iB_10104**	TC013627	TGCTGAAGAAGAGGCTCAAAATTTACGCAAGAAAGTGTCACGGATAGAAGATGATAATGAATCTTTGGTTTTGCAGCTGAAGAAAATGGCTACTCGAGCCAGAAGTCGTAAACTAAGTCCCACCAATAATTCAAGATTGACCCCAGAACCACCTTCTGAGAAAGGTGACGTATCAGACGACGAAGACCCCGCAGAAATCAAATTACAGTTAGAGTTGAGCGAACAAGAAGCTTCTGTTCTTCGACGCAAA
**iB_10181**	TC015547	GATGTACAGAGGGGGCAAGATCTGTCTCAGCGATCACTTCAAGCCTTTGTGGGCGCGAAACGTCCCCAAGTTTGGGATCGCGCACGCCATGGCGCTCGGGTTGGGGCCGTGGCTAGCCGTGGAAATCCCCGACTTGATCGCCAAAGGCGTGGTCACGCACAAGGAGAAA
**iB_10701**	TC014985	TCTCGTGTGTGCGTATCTCGCCAGAAATATATATTTTTATATTTATTTATGTTTTTGAAACAATAATTCTTCTATTTATCTAGTTAGGTTCGTTAGTGTAAAAATTGCTCAGTCATTATTTGTGTTTGTGACCACACACGTCCATAAACAGGCGAGTCTGC

bold: iBeetle annotated gland phenotype confirmed
